# Cost-effectiveness of granulocyte colony-stimulating factors (G-CSFs) for the prevention of febrile neutropenia (FN) in patients with cancer

**DOI:** 10.1007/s00520-023-08043-4

**Published:** 2023-09-20

**Authors:** Matti S. Aapro, Stephen Chaplin, Paul Cornes, Sebastian Howe, Hartmut Link, Natalia Koptelova, Andrea Mehl, Mario Di Palma, Bridgette Kanz Schroader, Robert Terkola

**Affiliations:** 1 Genolier Cancer Center, Genolier, Switzerland; 2 Xcenda UK, York, UK; 3 Comparative Outcomes Group, Bristol, UK; 4 Sandoz International GmbH, Industriestr. 18, D-83607 Holzkirchen, Germany; 5 Private Practice Hematology Oncology Kaiserslautern, Kaiserslautern, Germany; 6 Gustave Roussy, Paris-Saclay University, Villejuif, France; 7 Xcenda LLC, a Cencora company, Conshohocken, PA USA; 8 University Medical Center, Groningen, The Netherlands; 9 Paracelsus Medical University, Salzburg, Austria

**Keywords:** Febrile neutropenia, Cost-effectiveness, Filgrastim, Biosimilar, Pegfilgrastim

## Abstract

**Purpose:**

Clinical practice guidelines recommend the use of all approved granulocyte colony-stimulating factors (G-CSFs), including filgrastim and pegfilgrastim, as primary febrile neutropenia (FN) prophylaxis in patients receiving high- or intermediate-risk regimens (in those with additional patient risk factors). Previous studies have examined G-CSF cost-effectiveness by cancer type in patients with a high baseline risk of FN.

This study evaluated patients with breast cancer (BC), non-small cell lung cancer (NSCLC), or non-Hodgkin’s lymphoma (NHL) receiving therapy who were at intermediate risk for FN and compared primary prophylaxis (PP) and secondary prophylaxis (SP) using biosimilar filgrastim or biosimilar pegfilgrastim in Austria, France, and Germany.

**Methods:**

A Markov cycle tree-based model was constructed to evaluate PP versus SP in patients with BC, NSCLC, or NHL receiving therapy over a lifetime horizon. Cost-effectiveness was evaluated over a range of willingness-to-pay (WTP) thresholds for incremental cost per quality-adjusted life year (QALY) gained. Sensitivity analyses evaluated uncertainty.

**Results:**

Results demonstrated that using biosimilar filgrastim as PP compared to SP resulted in incremental cost-effectiveness ratios (ICERs) well below the most commonly accepted WTP threshold of €30,000. Across all three countries, PP in NSCLC had the lowest cost per QALY, and in France, PP was both cheaper and more effective than SP. Similar results were found using biosimilar pegfilgrastim, with ICERs generally higher than those for filgrastim.

**Conclusions:**

Biosimilar filgrastim and pegfilgrastim as primary prophylaxis are cost-effective approaches to avoid FN events in patients with BC, NSCLC, or NHL at intermediate risk for FN in Austria, France, and Germany.

**Supplementary Information:**

The online version contains supplementary material available at 10.1007/s00520-023-08043-4.

## Introduction

Febrile neutropenia (FN) is associated with significant morbidity in patients receiving myelosuppressive chemotherapy [1–4]. Mortality is also impacted through the direct health risks of FN and through the indirect effect of FN on delays and dose reductions of the patient’s active cancer treatment [5]. In addition, the costs and effects on hospital cancer services are significant. In the USA, FN is responsible for 5.2% of all cancer-related hospitalisations [6]. The risk and severity of FN depend on the chemotherapy regimen, dose intensity, and patient-specific risk factors [7]. Better prevention of FN could potentially benefit patients and healthcare systems at multiple levels.

Access to effective prevention of FN by granulocyte colony-stimulating factors (G-CSFs) is often limited by the high cost of these biologic medicines, resulting in restricted reimbursement by multiple health technology assessments (HTA) to only a subset of all the patients who could potentially benefit [8]. However, the loss of patent protection has enabled biosimilar versions of G-CSFs to be approved, creating competition to check prices. Simultaneously, real-world data on the performance of different formulations of G-CSFs have become available [9]. Together, this signals a need to reappraise the cost-effectiveness of traditional reimbursement decisions in this treatment space [10].

Clinical guidelines published by the European Society for Medical Oncology recommend the use of G-CSFs, including short-acting (filgrastim) and long-acting (pegfilgrastim) and approved biosimilars, as primary prophylaxis (PP) for FN in patients receiving high-risk regimens (> 20% risk of FN) or intermediate-risk regimens (> 10% risk) in the presence of additional patient risk factors [11]. Previously published studies have examined the cost-effectiveness of G-CSFs by cancer type; however, these have focused on patients with a high risk of FN [12–14].

In the USA, a study [15] evaluating the cost-effectiveness of G-CSFs in FN in patients receiving intermediate-risk first-line, curative-intent chemotherapy for breast cancer (BC), non-small cell lung cancer (NSCLC), or non-Hodgkin’s lymphoma (NHL) concluded that biosimilar filgrastim as PP compared to secondary prophylaxis (SP) provided an additional 0.102–0.144 life years (LY) and 0.065–0.130 quality-adjusted life years (QALY) at incremental costs ranging from $650–$2463 USD. To the best of our knowledge, no similar work has been published in Western Europe.

Based on this paucity of European data, the objective of this study was to build on the prior research in the USA to evaluate a similar European population of patients and compare PP and SP for FN using either the G-CSF biosimilar or respective reference product in patients at intermediate risk of FN. To represent the diversity of European healthcare, three different systems were included: Austria, with a two-tier system of centrally funded care and additional supplementary health insurance; France, with a centrally funded universal healthcare system; and Germany, with approximately 1100 public or private sickness funds with co-payments to prevent overutilisation and control costs [16].

## Methods

### Model structure

Building on the previously published cost-effectiveness model [15], a Markov cycle tree-based model was constructed in Microsoft^®^ Excel^®^ to evaluate the cost-effectiveness of PP versus SP with biosimilar filgrastim and biosimilar pegfilgrastim from the perspective of public health in Austria, France, and Germany [12, 13, 15].

**Fig. 1:**
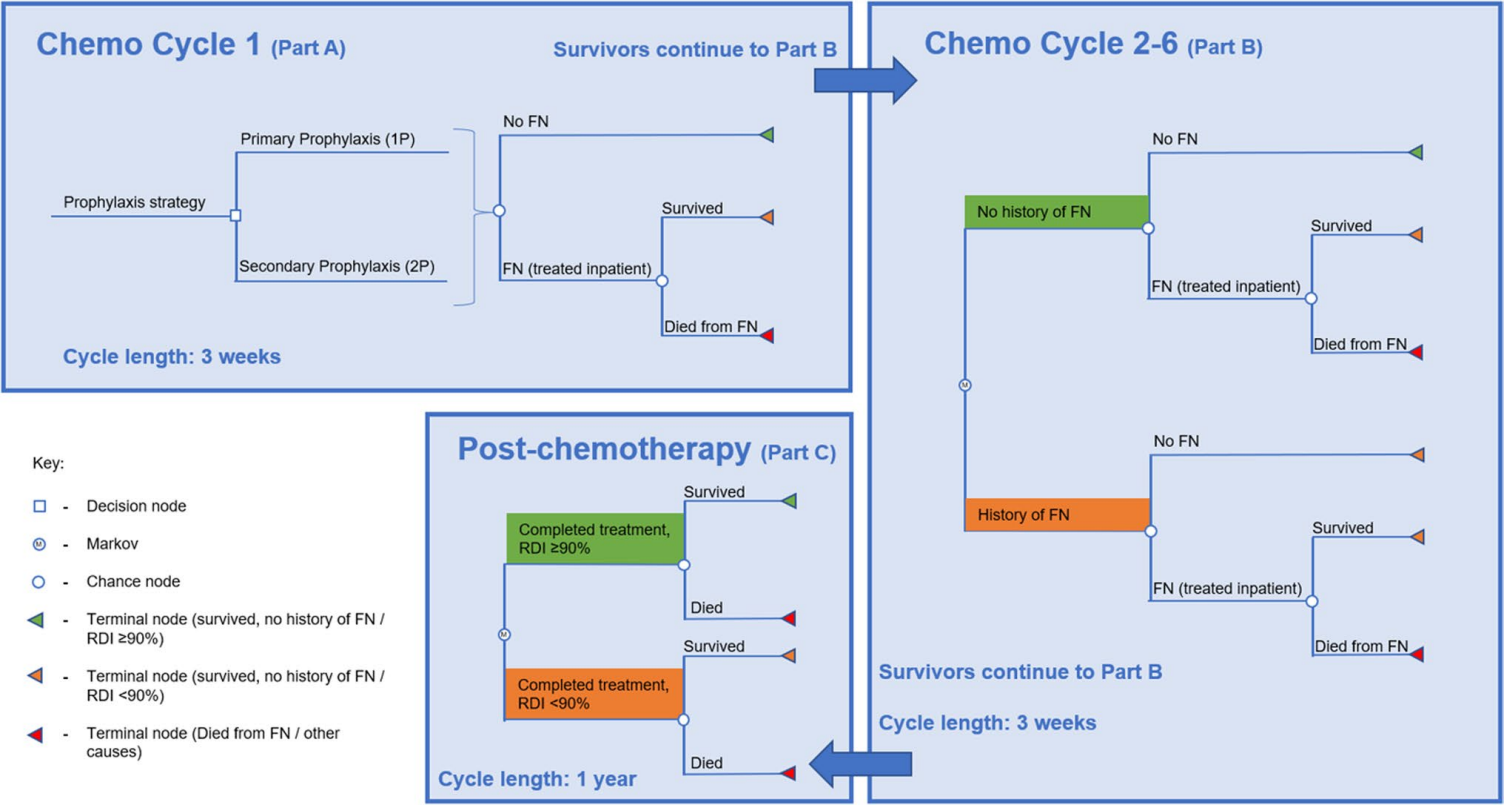
Model structure

The model structure is shown in Fig. 1 and was adapted to the specific number of cycles for each cancer type.

The first cycle was structured as a decision tree to pursue either a PP or SP strategy. The per-cycle risk of FN was estimated by distributing the baseline risk of FN over the full course of chemotherapy, with greater risk of FN in cycle 1 than subsequent cycles [17]. If an FN event occurred, all patients were treated in an inpatient setting and were at risk of dying or surviving to cycle 2 of chemotherapy. All cycle 1 deaths were assumed to be FN-related and occurred at the end of the cycle.

Chemotherapy cycles 2–6 consisted of a Markov cycle tree. In each 3-week cycle, patients entered with or without a history of FN. As in cycle 1, patients were at risk for FN, and if an FN event occurred, patients were treated as an inpatient and could survive to the next cycle or die from FN. All deaths in cycles 2–6 were assumed to be FN-related and to occur at the end of the cycle. Surviving patients repeated the Markov cycle until completing cycle 6. If patients had a history of FN from a prior cycle, the patient’s relative risk (RR) of FN would increase in subsequent cycles.

The post-chemotherapy phase consisted of 1-year Markov cycles. Initially, patients diverged into 2 groups based on cancer-specific risk considering their relative dose intensity (RDI) of chemotherapy and history of FN. Patients with a lower RDI were at higher annual risk of cancer-related death. Surviving patients re-entered the Markov cycle annually. After 20 years post-chemotherapy, mortality reverted to standard age- and sex-related rates as it was assumed patients were cured [18, 19]. The model considered a lifetime horizon.

To evaluate PP compared to SP with biosimilar filgrastim and biosimilar pegfilgrastim, incremental cost-effectiveness ratios (ICERs) were calculated for costs per FN event avoided, per LY gained, and per QALY gained. Cost-effectiveness was assessed at the commonly cited willingness-to-pay (WTP) threshold of €30,000 using SP as the reference comparator [20].

### Model parameters

Clinical, utility, and mortality inputs are presented in Table 1. In each analysis, the age at which patients entered the cohort varied according to their cancer type [4, 15]. Similarly, the baseline FN risks for each cancer type were selected to reflect the real world and focus on intermediate risk [4, 15].

**Table 1:** Clinical data

Parameter	Base-case value (range for PSA)^a^	Distribution
Baseline FN risk (over all cycles)^b^	BC: 0.158 (0.100–0.200) NSCLC: 0.180 (0.100–0.200) NHL: 0.180 (0.100–0.200)	*Beta*
RR of FN in cycles 2+, no history of FN (vs cycle 1)	0.21 (0.16–0.29)	*Lognormal*
RR of FN in cycles 2+, history of FN (vs no history)	9.09 (6.19–13.35)	*Lognormal*
RDI <*X*%, no history of FN	BC (85%): 0.309 (0.278–0.340) NSCLC (85%): 0.250 (0.225–0.275) NHL (90%): 0.408 (0.367–0.449)	*Beta*
RDI <*X*%, history of FN	BC (85%): 0.488 (0.371–0.649) NSCLC (85%): 0.383 (0.345–0.421) NHL (90%): 0.706 (0.635–0.777)	*Beta*
RR of FN for filgrastim (vs no CSF)	0.42 (0.30–0.57)	*Lognormal*
Health utility inputs		
During chemotherapy	BC: 0.55 (0.50–0.61) NSCLC: 0.57 (0.51-0.63) NHL: 0.61 (0.49–0.73)	*Beta*
During FN hospitalisation	0.33 (0.27–0.40)	*Beta*
After chemotherapy (year 1)	BC: 0.66 (0.59–0.73) NSCLC: 0.72 (0.65–0.79) NHL: 0.79 (0.62–0.92)	*Beta*
After chemotherapy (year >1)	BC: 0.86 (0.77–0.95) NSCLC: 0.69 (0.62–0.76) NHL: 0.89 (0.79–0.96)	*Beta*
Mortality inputs		
Cancer-related 1-year mortality^c^	BC: 0.0300 (0.0270–0.0330) NSCLC: 0.0600 (0.0540–0.0660) NHL: 0.0652 (0.0587–0.0717)	*Beta*
Mortality during FN event (inpatient)	BC: 0.0560 (0.0480–0.0630) NSCLC: 0.1120 (0.1010–0.1230) NHL: 0.0580 (0.0000–0.0890)	*Beta*
HR for mortality, RDI <*X*% (vs RDI ≥*X*%)	BC (85%): 1.002 (0.657–1.527) NSCLC (85%): 2.004 (1.159–3.463) NHL (90%): 2.080 (1.190–3.700)	*Lognormal*

Clinical input references are cited in previously published model by Li et al. [15]

*BC* breast cancer, *CHOP* cyclophosphamide, doxorubicin, vincristine, and prednisone, *CSF* colony-stimulating factor, *FN* febrile neutropenia, *HR* hazard ratio, *LOS* length of stay, *NHL* non-Hodgkin lymphoma, *NSCLC* non-small cell lung cancer, *PP* primary prophylaxis, *RDI* relative dose intensity, *RR* relative risk, *SP* secondary prophylaxis

^a^Unless otherwise specified, the parameter values presented apply to all 3 models

^b^For BC, values are based on the FN rate for docetaxel every 3 weeks. For NSCLC, value is based on the FN risk over the treatment course in patients receiving carboplatin and paclitaxel for non-metastatic non-small cell lung cancer and with ≥ 1 risk factor for FN. In the analysis, 97.3% of such patients had ≥ 1 risk factor and only 12.3% received CSF prophylaxis in the first cycle. For NHL, value is based on the cumulative probability of FN over 126 days of CHOP therapy for patients with 2 risk factors

^c^For BRCA and NHL, value is based on % survival over 5 years with the respective cancer, and the 1-year probability for death was calculated by first converting the 5-year probability to the instantaneous rate using the following equation: *r* = -[ln(1-P)]/*t*

Disease-specific chemotherapy regimens associated with intermediate risk of FN were considered including docetaxel and cyclophosphamide (TC) in BC; rituximab, cyclophosphamide, doxorubicin, vincristine, and prednisolone (R-CHOP) in NHL; and carboplatin and paclitaxel in NSCLC. Base-case inputs for the effectiveness of filgrastim and pegfilgrastim versus no G-CSF in reducing the risk of FN per cycle were based on a meta-analysis evaluating trials of G-CSF in solid tumours by cancer type [14]. The RR of FN for filgrastim was 0.42; for pegfilgrastim, 0.25; these were assumed to be the same for BC, NSCLC, and NHL [15, 21].

As the risk for FN over the entire course of chemotherapy is greatest in the first cycle, baseline cycle-specific FN risk was calculated as the risk in patients without a history of FN and without G-CSF prophylaxis. This baseline risk was decreased in patients who received G-CSF prophylaxis [12, 15]. History of FN increased the likelihood of subsequent FN events [15].

Health benefits in QALYs were calculated by adjusting LYs with utility values for, during, and after chemotherapy. Utility values were sourced from previously published cost-effectiveness analyses [12, 13, 15]. A hospitalisation for FN was expected to have a significant impact on quality of life while receiving chemotherapy. After all cycles of chemotherapy are complete, quality of life for the first year was expected to improve, with greater improvement after 1-year post-chemotherapy [12, 13, 15, 22–24]. Post-chemotherapy utility was assumed to remain constant until death.

Although long-term cancer survival rates vary across the three cancer types, long-term relapse rates are not influenced by G-CSF usage. Therefore, mortality rates over 1 year were based on 5-year survival rates adjusted to a 1-year probability of death for all cancer types. The model assumed the cancer-specific mortality rate was additive to the age- and sex-specific standard mortality rates for each country and assumed to only apply during the first 20 years following chemotherapy, after which the patient reverted to the standard country-specific age- and sex-specific mortality rates, as previously described [12].

A summary of cost inputs in the model is shown in Online Resource 1. Costs included in the model were treatment with biosimilar filgrastim and biosimilar pegfilgrastim and inpatient FN management. The cost of G-CSF administration was assumed to be included in the cost of inpatient admission. Chemotherapy costs were excluded from the analysis as they were assumed to be equivalent between patients receiving PP and SP. Similarly, the difference in chemotherapy costs for patients receiving low versus high RDI was assumed to be negligible. Post-chemotherapy costs were assumed to be unaffected by prophylaxis strategy and were therefore excluded. Drug costs were based on published list price information per country for a representative biosimilar filgrastim and representative biosimilar pegfilgrastim.

### Sensitivity analysis

Alternative parameter values were tested via a one-way sensitivity analysis (OWSA) to evaluate the impact of each parameter on the models’ outcomes. In addition, a probabilistic sensitivity analysis (PSA) accounted for joint uncertainty among all model parameters and assessed the likelihood of cost-effectiveness of PP over a range of WTP thresholds. The PSA simulated 1000 iterations with parameter values sampled simultaneously from their individual distributions.

## Results

Results are presented separately by cancer, country, and filgrastim/pegfilgrastim in Table 2 (Austria), Table 3 (France), and Table 4 (Germany) and are summarised as follows.

**Table 2:** Base-case results by cancer type—Austria

Comparator	Costs	FN events avoided	LYs	QALYs	ICER (€/FN event avoided)	ICER (€/LY)	ICER (€/QALY)
Base-case results: BC—biosimilar filgrastim							
Primary prophylaxis	€1567	0.135	13.024	10.997	€5840	€8029	€9219
Secondary prophylaxis	€980	0.035	12.951	10.934	Reference	Reference	Reference
Base-case results: NSCLC—biosimilar filgrastim							
Primary prophylaxis	€1847	0.156	8.743	6.023	€972	€758	€1069
Secondary prophylaxis	€1736	0.041	8.597	5.919	Reference	Reference	Reference
Base-case results: NHL—biosimilar filgrastim							
Primary prophylaxis	€2506	0.172	8.348	7.266	€5459	€4195	€4646
Secondary prophylaxis	€1862	0.054	8.194	7.127	Reference	Reference	Reference
Base-case results: BC—biosimilar pegfilgrastim							
Primary prophylaxis	€1787	0.168	13.099	11.063	€6724	€9205	€10,568
Secondary prophylaxis	€959	0.045	13.009	10.984	Reference	Reference	Reference
Base-case results: NSCLC—biosimilar pegfilgrastim							
Primary prophylaxis	€1957	0.194	8.791	6.057	€2036	€1558	€2199
Secondary prophylaxis	€1672	0.054	8.608	5.927	Reference	Reference	Reference
Base-case results: NHL—biosimilar pegfilgrastim							
Primary prophylaxis	€2751	0.212	8.397	7.310	€6781	€4926	€5458
Secondary prophylaxis	€1786	0.070	8.201	7.133	Reference	Reference	Reference

*BC* breast cancer, *FN* febrile neutropenia, *ICER* incremental cost-effectiveness ratio, *NHL* non-Hodgkin’s lymphoma, *NSCLC* non-small cell lung cancer, *QALY* quality-adjusted life year

**Table 3:** Base-case results by cancer type—France

Comparator	Costs	FN events avoided	LYs	QALYs	ICER (€/FN event avoided)	ICER (€/LY)	ICER (€/QALY)
Base-case results: BC—biosimilar filgrastim							
Primary prophylaxis	€1880	0.135	12.943	10.928	€4489	€6211	€7131
Secondary prophylaxis	€1430	0.035	12.870	10.865	Reference	Reference	Reference
Base-case results: NSCLC—biosimilar filgrastim							
Primary prophylaxis	€2323	0.156	8.272	5.697	Dominates^a^	Dominates	Dominates
Secondary prophylaxis	€2475	0.041	8.134	5.599	Reference	Reference	Reference
Base-case results: NHL—biosimilar filgrastim							
Primary prophylaxis	€3165	0.172	7.974	6.933	€6421	€5248	€5807
Secondary prophylaxis	€2407	0.054	7.829	6.802	Reference	Reference	Reference
Base-case results: BC—biosimilar pegfilgrastim							
Primary prophylaxis	€2406	0.168	13.014	10.990	€8039	€11,076	€12,715
Secondary prophylaxis	€1416	0.045	12.925	10.912	Reference	Reference	Reference
Base-case results: NSCLC—biosimilar pegfilgrastim							
Primary prophylaxis	€2656	0.194	8.316	5.729	€1839	€1499	€2112
Secondary prophylaxis	€2398	0.054	8.144	5.607	Reference	Reference	Reference
Base-case results: NHL—biosimilar pegfilgrastim							
Primary prophylaxis	€3656	0.212	8.020	6.974	€9358	€7233	€8008
Secondary prophylaxis	€2325	0.070	7.836	6.808	Reference	Reference	Reference

*BC* breast cancer, *FN* febrile neutropenia, *ICER* incremental cost-effectiveness ratio, *NHL* non-Hodgkin’s lymphoma, *NSCLC* non-small cell lung cancer, *QALY* quality-adjusted life year

^a^Primary prophylaxis is cheaper and more effective

**Table 4:** Base-case results by cancer type—Germany

Comparator	Costs	FN events avoided	LYs	QALYs	ICER (€/FN event avoided)	ICER (€/LY)	ICER (€/QALY)
Base-case results: BC—biosimilar filgrastim							
Primary prophylaxis	€2411	0.135	13.812	11.675	€18,781	€24,334	€27,960
Secondary prophylaxis	€525	0.035	13.734	11.608	Reference	Reference	Reference
Base-case results: NSCLC—biosimilar filgrastim							
Primary prophylaxis	€2554	0.156	8.650	5.959	€13,550	€10,698	€15,090
Secondary prophylaxis	€1003	0.041	8.505	5.856	Reference	Reference	Reference
Base-case results: NHL—Biosimilar filgrastim							
Primary prophylaxis	€3904	0.172	8.397	7.310	€20,909	€15,878	€17,588
Secondary prophylaxis	€1437	0.054	8.242	7.169	Reference	Reference	Reference
Base-case results: BC—biosimilar pegfilgrastim							
Primary prophylaxis	€4077	0.168	13.889	11.742	€27,862	€35,951	€41,305
Secondary prophylaxis	€646	0.045	13.794	11.659	Reference	Reference	Reference
Base-case results: NSCLC—biosimilar pegfilgrastim							
Primary prophylaxis	€4181	0.194	8.697	5.992	€21,932	€17,003	€23,990
Secondary prophylaxis	€1108	0.054	8.517	5.864	Reference	Reference	Reference
Base-case results: NHL—biosimilar pegfilgrastim							
Primary prophylaxis	€6252	0.212	8.447	7.354	€32,746	€23,499	€26,046
Secondary prophylaxis	€1594	0.070	8.248	7.175	Reference	Reference	Reference

*BC* breast cancer, *FN* febrile neutropenia, *ICER* incremental cost-effectiveness ratio, *NHL* non-Hodgkin’s lymphoma, *NSCLC* non-small cell lung cancer, *QALY* quality-adjusted life year

### Austria

Base-case results for Austria show that across the three cancers, biosimilar filgrastim as PP versus SP provided an additional 0.073–0.154 LYs and 0.063–0.139 QALYs and prevented 0.100–0.118 FN events. The highest gains were for patients with NHL and the lowest gains were for patients with BC. Incremental cost ranged from €111–€644. The ICERs ranged from €972–€5840 per FN event avoided, €758–€8029 per LY gained, and €1069–€9219 per QALY gained, with NSCLC reflecting the lowest ICERs.

According to the OWSA for cost per QALY gained (Online Resource 2), for NSCLC, variation in baseline risk of FN, mean hospital length of stay (LOS) for FN, and RR of FN for filgrastim had the greatest influence on the results. For BC and NHL, variation in baseline risk of FN, mortality hazard ratio for RDI, and RR of FN for filgrastim were the most influential.

For pegfilgrastim, incremental LY gains, QALY gains, and FN events avoided were slightly higher than for filgrastim (0.090–0.196 LYs and 0.079–0.177 QALYs gained and 0.123–0.142 FN events prevented). As with filgrastim, the highest gains were for patients with NHL, and the lowest gains were for patients with BC. Incremental costs were higher than those for filgrastim, ranging from €285–€965. The ICERs ranged from €2036–€6781 per FN event avoided, €1558–€9205 per LY gained, and €2199–€10,568 per QALY gained, with NSCLC reflecting the lowest ICERs.

According to the OWSA on the cost per QALY gained, for NSCLC variation in baseline risk of FN, mean length of stay for a hospitalisation and RR of FN for pegfilgrastim had the greatest influence on the results. For BC variation in baseline risk of FN, mortality hazard ratio of RDI and discount rate had the greatest influence on the results; for NHL, the inputs with the greatest influence were baseline risk of FN, the mortality hazard ratio of RDI, and RR of FN for pegfilgrastim.

### France

Base-case results for France show that across the three cancers, biosimilar filgrastim as PP versus SP provided an additional 0.073–0.145 LYs and 0.063–0.131 QALYs. As efficacy inputs are the same across all three countries, FN events prevented were the same as those for Austria. QALY and LY results differed from those in Austria due to country-specific mortality data. The highest gains were for patients with NHL and lowest were for patients with BC. Incremental costs ranged from savings of €152 for NSCLC to €758 for NHL. The ICERs for NHL and BC were €6421 and €4489 per FN event avoided, €5248 and €6211 per LY gained, and €5807 and €7131 per QALY gained, respectively. For NSCLC, PP was both cheaper and more effective than SP and therefore PP dominated SP.

According to the OWSA for cost per QALY gained (Online Resource 3), for NSCLC variation in baseline risk of FN, mean LOS when hospitalised for FN and RR of FN for filgrastim were the only inputs that resulted in PP not dominating SP. For BC variation in baseline risk of FN, mortality hazard ratio for RDI and RR of FN for filgrastim had the greatest influence on the results. For NHL, the inputs with the greatest influence were baseline risk of FN, RR of FN for filgrastim, and the patient’s weight.

For pegfilgrastim, PP provided an additional 0.089–0.184 LYs and 0.078–0.166 QALYs. The highest gains were for patients with NHL, and lowest were in patients with BC. Incremental costs ranged from €258–€990. The ICERs ranged from €1839–€9358 per FN event avoided, €1499–€11,076 per LY gained, and €2112–€12,715 per QALY gained, with NSCLC reflecting the lowest ICERs.

According to the OWSA for cost per QALY gained, for NSCLC variation in baseline risk of FN, mean LOS for a hospitalisation and RR of FN for pegfilgrastim had the greatest influence on the results. For BC and NHL variation in baseline risk of FN, the mortality hazard ratio of RDI and RR of FN for pegfilgrastim had the greatest influence on the results.

### Germany

Base-case results for Germany show that across the three cancers, biosimilar filgrastim as PP versus SP provided an additional 0.078–0.145 LYs and 0.067–0.141 QALYs. The highest gains were for patients with NHL and lowest were in patients with BC. Incremental costs were higher than in both Austria and France, ranging from €1551–€1886. Due to these higher incremental costs, ICERs in Germany are higher for all three cancer types. The cost per FN event avoided ranged from €13,550–€20,909, the cost per LY gained ranged from €10,698–€24,334, and the cost per QALY gained ranged from €15,090–€27,960, with NSCLC reflecting the lowest ICERs.

Similar to Austria and France, the OWSA showed that for NSCLC variation in baseline risk of FN (Online Resource 4), RR of FN for filgrastim and cost of an FN event hospitalisation had the greatest influence on results. For BC variation in baseline risk of FN, the mortality hazard ratio for RDI and the discount rate had the greatest influence on results. For NHL, the inputs with the greatest influence were baseline risk of FN, RR of FN for filgrastim, and the patient’s weight.

For pegfilgrastim, PP provided an additional 0.095–0.199 LYs and 0.083–0.179 QALYs. The highest gains were for patients with NHL and the lowest were in patients with BC. Incremental costs ranged from €3073–€4658. The ICERs ranged from €21,932–€32,746 per FN event avoided, €17,003–€35,951 per LY gained, and €23,990–€41,305 per QALY gained, with NSCLC reflecting the lowest ICERs. As for filgrastim, ICERs were higher in Germany than in Austria and France

According to the OWSA on the cost per QALY gained, for NSCLC variation in baseline risk of FN, RR of FN for pegfilgrastim and the discount rate had the greatest influence on the results. For BC and NHL variation in baseline risk of FN, the mortality hazard ratio of RDI and RR of FN for pegfilgrastim had the greatest influence on the results.

### PSA results

The PSA identified that for filgrastim, at a WTP threshold of €50,000 per QALY gained, the probability of the cost-effectiveness of PP remained high across all three cancer types for all three countries (ranging from 95%–100%). Similar results were shown for pegfilgrastim (96%–100%).

## Discussion

Europe has 18 different versions of filgrastim or pegfilgrastim, either as originator or as biosimilar brand [25]. This has been sufficient to change cost-effectiveness assumptions behind European guidelines—as this study shows. American studies are in agreement, confirming that while the costs of the FN events remain high, lower drug costs support the concept that PP may be cost-effective at even modest spending thresholds [26–28].

Based on this analysis, expanding reimbursement to cover biosimilar filgrastim or pegfilgrastim for patients treated with intermediate-risk chemotherapy regimens would be considered cost-effective. This is an important consideration also in view of the present COVID crisis and appeals of learned societies for a more liberal use of preventive growth factors [29]. It resulted in ICERs well below the commonly used WTP threshold of €30,000. Across all three countries, NSCLC had the lowest cost per QALY, and in France, PP dominated SP such that PP was considered both cheaper and more effective. Similar results were found using pegfilgrastim, with ICERs generally higher than those for filgrastim.

This base-case assessment used current ex-manufacturer list prices, which is common practice when undertaking an HTA. In Austria, however, pricing is dependent on whether a drug is prescribed at the initial clinic visit, when treatment is covered under a disease-related group payment, or after this when the pharmacy sale list price is considered. Scenario analysis at the upper limit pricing for filgrastim (€55.54 per 300 mcg) and pegfilgrastim (€430.75 per dose) showed that PP remains cost-effective across all three cancer types for both filgrastim and pegfilgrastim. In France, tendering is common practice. As such, prices are likely to be lower than the list price used in the base case. Scenario analysis was performed for a 30% discount for biosimilar filgrastim (€40) and 15% for biosimilar pegfilgrastim (€400); as expected given a lower price, PP remains cost-effective. Importantly, past experience suggests that real-world costs will fall and cost-effectiveness will rise still further as the competitive market for medicines created by biosimilars creates further tender-based discounts [30].

The value of G-CSFs changes over time as research identifies patient groups at greater chance of clinical benefit while prices can change due to competition [8]. To remain relevant, guidelines based around cost-effectiveness estimates, such as the current US and European guidelines for G-CSF use, must also change over time. The conventional perception has been that G-CSFs are traditionally expensive drugs that should be conserved for perceived high-value patient scenarios, e.g. patients at high risk of FN, who theoretically incur higher incidence rates and subsequent cost burdens with episodes of FN. This perception is now challenged by the findings that biosimilars deliver significant cost savings related to the number of competing brands and time since biosimilar launches in both Europe and the USA [31, 32], with the focus of treatment changing to ensuring that guidelines are met [33, 34].

Furthermore, previous studies of biosimilar treatments in Europe have shown that the benefits of biosimilars are not limited to costs savings and may expand access to treatment and improve cost-effectiveness of the treatment [35]. The results of the modelling presented here have shown similar benefits in the use of biosimilar treatments.

There are some limitations to this study. First, the structure of the analyses likely represents a simplification of the complex interplay between disease, treatments, and costs. For example, our model considered the FN risk associated with the regimen but was unable to consider the FN risk of individual patients as we were limited by available data inputs. Some patients may have been treated in the outpatient setting based on individual risk factors instead of in the hospital, which would likely be less costly. However, multiple studies have suggested that, while patient-specific risk scoring systems exist, most patients who present to emergency departments with FN are admitted; thus, the potential cost savings for outpatient management are minimal as outpatient management is not yet common practice [36, 37]. Future models could consider patient-specific risk factors. Next, for BC, a TC treatment regimen was assumed [38]. There is some debate in the USA as to whether this regimen is associated with intermediate or high risk of FN [39]. However, in a European setting, the prophylactic strategy commonly employed assumes it is associated with an intermediate risk of FN based on clinical studies reporting an associated FN risk of 15.8% [40], and, as such, that was how it was incorporated into this model. In the previous USA study [15], patients with BC received a taxane (docetaxel) regimen, with an associated baseline FN risk of 16% [41], an intermediate-risk regimen.

Our study reports data for only three countries, confirming a consistent impact of biosimilars across a diversity of European health systems, but it is important to note that the results of this model reflect current practice managing these patient populations only in the countries we have included (France, Germany, and Austria). For these results and potential cost savings to be applicable to other countries, the model would have to be recalculated. For example, published literature in the UK [42] and Australia [43] have reported successful management of patients with BC and low-risk FN in an ambulatory setting and, in the study in Australia, cost savings with this approach. Management of patients in an ambulatory setting would likely result in a lower estimate of cost savings than in our present model. By publishing the details of our model, the cost-effectiveness of expanding reimbursement to cover intermediate-risk chemotherapy regimens can be estimated using HTAs for other health systems, including systems for which transparent costs are not publicly available.

In the absence of data for each cancer type, some inputs were based on studies that examined patients with different cancer types, including the RR of an FN event where it was assumed that the RR for NHL and NSCLC was the same as for BC and self-administration of G-CSF was assumed to be the same across all cancers in the absence of cancer-specific data. Adverse events associated with G-CSFs were not included in the model. The most common adverse event of G-CSFs is bone pain, followed by headache, nausea/vomiting, fever/chills, fatigue, skin reaction, and myalgias, all of which are managed with inexpensive medications that would not add significant costs to the model [44–47]. Utility values in this model were based on data obtained from clinicians, as direct patient utility data for FN were not available. Finally, the model assumed that all FN events required a hospital admission. FN-related events that occurred outside of the hospital setting were not captured.

The comparability of our findings across three such different health systems suggests that to stay relevant, all HTAs and their associated value-based guidelines will need to undergo regular re-assessments after biosimilars have been launched. The European Commission plans to harmonise HTA methodology in a few years. This suggests that such a rolling programme of post-biosimilar assessment could quickly become routine as a shared resource across a wide range of guideline groups and different national health systems.

## Conclusion

Based on this analysis, using biosimilar filgrastim and pegfilgrastim as PP is a cost-effective approach to avoid FN events across all three cancer types and for all three countries, with NSCLC being the most cost-effective. This information suggests there is no rationale to justify additional restrictions to treatment guidelines which exist in some European countries with high costs.

### Supplementary Information

ESM 1 (PDF 109 kb)

ESM 2 (PDF 185 kb)

ESM 3 (PDF 218 kb)

ESM 4 (PDF 221 kb)
