# Supplementary material for: Cost-effectiveness of granulocyte colony-stimulating factors (G-CSFs) for the prevention of febrile neutropenia (FN) in patients with cancer
Source: Support Care Cancer. 2023 Sep 20;31(10):581. doi: 10.1007/s00520-023-08043-4 (PMC10511548; doi:10.1007/s00520-023-08043-4)
Supplement: Supplementary file 1 — (PDF 109 kb) [file 520_2023_8043_MOESM1_ESM.pdf]

## TITLE PAGE

Cost -effectiveness of granulocyte colony stimulating factors (G-CSFs) for the prevention of febrile neutropenia (FN) in patients with cancer

Authors: Matti S. Aapro[a], MD, Stephen Chaplin[b], BSc, Paul Cornes[c], BM; Sebastian Howe[d], PhD, Hartmut Link[e], MD, PhD, Natalia Koptelova[d], MD, Andrea Mehl[d], MBA, MPHEco, Mario Di Palma[f], MD, Bridgette Kanz Schroader[g], PharmD, MPA, BCOP, Robert Terkola[h,i], PhD, MSc, aHPh; .

Affiliations: [a] Genolier Cancer Center, Genolier, Switzerland, [b] Xcenda UK, York, [c] Comparative Outcomes Group, UK, [d] Sandoz International GmbH, Holzkirchen, Germany, [e] Private Practice Hematology Oncology Kaiserslautern, Germany, [f] Gustave Roussy, Paris-Saclay University, Villejuif, France, [g] Xcenda LLC, Carrollton, TX, US, [h] Institute of Science in Healthy Ageing & HealthcarE (SHARE, University Medical Center Groningen (UMCG, University of Groningen, The Netherlands), [i] Department of Pharmacotherapy and Translational Research, University of Florida -College of Pharmacy, Gainesville, USA

**Corresponding author:**

Dr Sebastian Howe

Sandoz International GmbH

Industriestr. 18

D-83607 Holzkirchen

Germany

Telephone: +49 8024 4763508

Email address: sebastian.howe@sandoz.com

## Online resource 1: Cost inputs

| | **Breast Cancer** | **NHL** | **NSCLC** |
|---|---|---|---|
| **Austria** | | | |
| Filgrastim-sndz (price per mcg) [1] | €0.16 | €0.16 | €0.16 |
| Pegfilgrastim-bmez [1] | €390.86 | €390.86 | €390.86 |
| Cost of FN event requiring hospitalization (per day) [2] | €923.00 | €923.00 | €923.00 |
| Number of filgrastim doses per chemotherapy cycle [3] | 5 | 5 | 5 |
| Number of pegfilgrastim doses per chemotherapy cycle | 1 | 1 | 1 |
| Percentage of patients self-administering G-CSF [4] | 34% | 34% | 34% |
| Percentage of FN events requiring hospitalisation (Assumption) | 100% | 100% | 100% |
| Mean length of stay for FN - PP | 5.7 [5,6] | 8.8 [7] | 8.8 [7] |
| Mean length of stay for FN - SP | 5.4 [5,6] | 8.8 [7] | 8.8 [7] |
| **France** | | | |
| Filgrastim-sndz (cost per mcg) [8] | €0.20 | €0.20 | €0.20 |
| Pegfilgrastim-bmez [8] | €521.73 | €521.73 | €521.73 |
| Cost of FN event requiring hospitalization (per day) [9] | €1,317 | €1,317 | €1,317 |
| Number of filgrastim doses per chemotherapy cycle[3] | 5 | 5 | 5 |
| Number of pegfilgrastim doses per chemotherapy cycle | 1 | 1 | 1 |
| Percentage of patients self-administering G-CSF[4] | 18% | 18% | 18% |
| Percentage of FN events requiring hospitalisation (assumption) | 100% | 100% | 100% |
| Mean length of stay for FN - PP [10] | 5.7 | 8.0 | 8.9 |
| Mean length of stay for FN - SP [10] | 5.7 | 8.0 | 8.9 |
| **Germany** | | | |
| Filgrastim-sndz (cost per mcg) [11] | €0.32 | €0.32 | €0.32 |
| Pegfilgrastim-bmez [11] | €998.75 | €998.75 | €998.75 |
| Cost of FN event requiring hospitalization (per day) [7] | €355.78 | €606.46) | €457.50 |
| Number of filgrastim doses per chemotherapy cycle[3] | 5 | 5 | 5 |
| Number of pegfilgrastim doses per chemotherapy cycle | 1 | 1 | 1 |
| Percentage of patients self-administering G-CSF[4] | 34% | 34% | 34% |
| Percentage of FN events requiring hospitalisation (assumption) | 100% | 100% | 100% |
| Mean length of stay for FN - PP | 5.7[5,6] | 8.8 [7] | 8.8[7] |
| Mean length of stay for FN - SP | 5.4[5,6] | 8.8 [7] | 8.8 [7] |
| Abbreviations: FN, febrile neutropenia; G-CSF, Granulocyte colony stimulating factor; mcg, micrograms; PP, primary prophylaxis; SP, secondary prophylaxis.<br>References: | | | |

1. Warenverzeichnis. Österreichische Apotheker-Verlagsgesellschaft m.b.H. 2021, Vienna, Austria. 2021.
2. Austrian Ministry of Social Affairs, Health, Care and Consumer Protection. http://www.kaz.bmgf.gv.at/kosten.html
3. Gascon P, Aapro M, Ludwig H, et al. Treatment patterns and outcomes in the prophylaxis of chemotherapy-induced (febrile) neutropenia with biosimilar filgrastim (the MONITOR-GCSF study). *Support Care Cancer*. Feb 2016;24(2):911-925. doi:10.1007/s00520-015-2861-z
4. Sandoz. Data on file. 2021;
5. Jacobs VR, Mayer SC, Paessens BJ, et al. Comparison of Actual Hospital Costs versus DRG Revenues for In-Patient Treatment of Febrile Neutropenia during Adjuvant Anthracycline plus/minus Taxane-Based Chemotherapy for Primary Breast Cancer. *Oncology Research and Treatment*. 2011;34(11):614-618. doi:10.1159/000334063
6. Renner P, Milazzo S, Liu JP, Zwahlen M, Birkmann J, Horneber M. Primary prophylactic colony-stimulating factors for the prevention of chemotherapy-induced febrile neutropenia in breast cancer patients. *Cochrane Database Syst Rev*. Oct 17 2012;10:Cd007913. doi:10.1002/14651858.CD007913.pub2
7. Ihbe-Heffinger A, Paessens BJ, von Schilling C, et al. Management of febrile neutropenia--a German prospective hospital cost analysis in lymphoproliferative disorders, non-small cell lung cancer, and primary breast cancer. *Onkologie*. 2011;34(5):241-6. doi:10.1159/000327711
8. Ameli l'Assurance Maladie. www.ameli.fr
9. Liste des tarifs applicables à l'AP-HP. "https://pitiesalpetriere.aphp.fr/wp-content/blogs.dir/58/files/2019/03/Affiche-tarifs-mars2019-2.pdf"
10. Freyer G, Scotte F, Borget I, Bruyas A, Vainchtock A, Chouaid C. [Clinical burden caused by hospitalization for febrile neutropenia in France in 2010-2011: An analysis of the PMSI database]. *Bull Cancer*. Jun 2016;103(6):552-60. Hospitalisations pour neutropenie febrile chimio-induite en France en 2010-2011 : impact clinique et caracteristiques des patients a partir des donnees de la base PMSI. doi:10.1016/j.bulcan.2016.03.012
11. Lauer Taxe. https://www.cgm.com/deu_de/produkte/apotheke/lauer-taxe.html
