# Supplementary material for: Cost-effectiveness of granulocyte colony-stimulating factors (G-CSFs) for the prevention of febrile neutropenia (FN) in patients with cancer
Source: Support Care Cancer. 2023 Sep 20;31(10):581. doi: 10.1007/s00520-023-08043-4 (PMC10511548; doi:10.1007/s00520-023-08043-4)
Supplement: Supplementary file 2 — (PDF 185 kb) [file 520_2023_8043_MOESM2_ESM.pdf]

Title: Cost -effectiveness of granulocyte colony stimulating factors (G-CSFs) for the prevention of febrile neutropenia (FN) in patients with cancer

Authors: Matti S. Aapro[a], MD, Stephen Chaplin[b], BSc, Paul Cornes[c], BM; Sebastian Howe[d], PhD, Hartmut Link[e], MD, PhD, Natalia Koptelova[d], MD, Andrea Mehl[d], MBA, MPHEco, Mario Di Palma[f], MD, Bridgette Kanz Schroader[g], PharmD, MPA, BCOP, Robert Terkola[h,i], PhD, MSc, aHPh.

Affiliations: [a] Genolier Cancer Center, Genolier, Switzerland; [b] Xcenda UK, York; [c] Comparative Outcomes Group, UK; [d] Sandoz International GmbH, Holzkirchen, Germany; [e] Private Practice Hematology Oncology Kaiserslautern, Germany; [f] Gustave Roussy, Paris-Saclay University, Villejuif, France; [g] Xcenda LLC, Carrollton, TX, US; [h] Institute of Science in Healthy Ageing & HealthcarE (SHARE, University Medical Center Groningen (UMCG, University of Groningen, The Netherlands); [i] Department of Pharmacotherapy and Translational Research, University of Florida -College of Pharmacy, Gainesville, USA

Corresponding author:

Dr Sebastian Howe

Sandoz International GmbH

Industriestr. 18

D-83607 Holzkirchen

Germany

Telephone: +49 8024 4763508

Email address: sebastian.howe@sandoz.com

Online Resource 2: Austria OWSA

Abbreviations: FN – febrile neutropenia; G-CSF – granulocyte colony stimulating factor; ICER – incremental cost-effectiveness ratio; kg – kilogram; LOS – length of stay; mcg – microgram; NHL – non-Hodgkin’s lymphoma; NSCLC – non-small cell lung cancer; OWSA – one-way sensitivity analysis; QALY – quality-adjusted life year; RDI – relative dose intensity; SP – secondary prophylaxis.

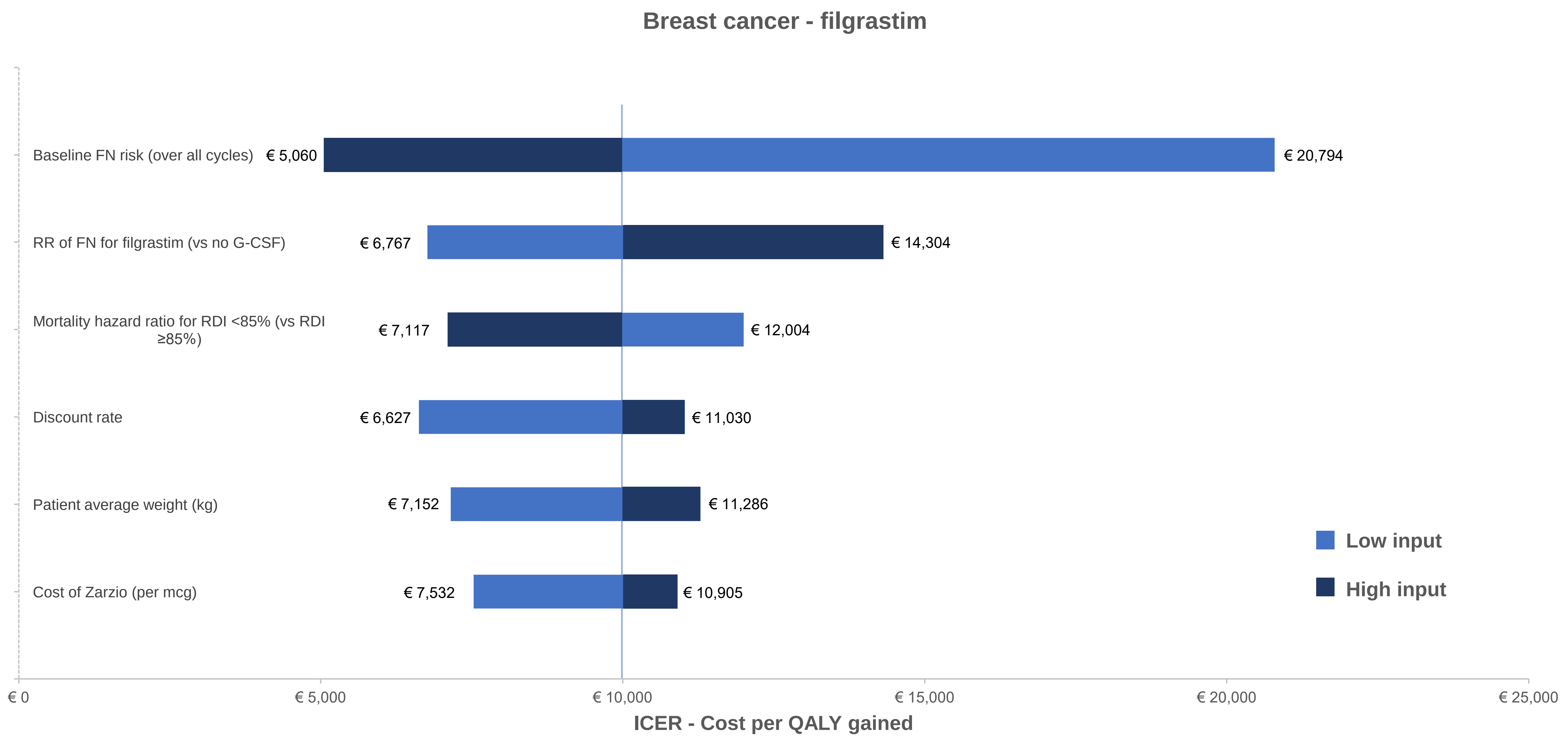
Breast cancer - filgrastim
Baseline FN risk (over all cycles)
€ 5,060
€ 20,794
RR of FN for filgrastim (vs no G-CSF)
€ 6,767
€ 14,304
Mortality hazard ratio for RDI <85% (vs RDI ≥85%)
€ 7,117
€ 12,004
Discount rate
€ 6,627
€ 11,030
Patient average weight (kg)
€ 7,152
€ 11,286
Cost of Zarzio (per mcg)
€ 7,532
€ 10,905
Low input
High input
€ 0
€ 5,000
€ 10,000
€ 15,000
€ 20,000
€ 25,000
ICER - Cost per QALY gained

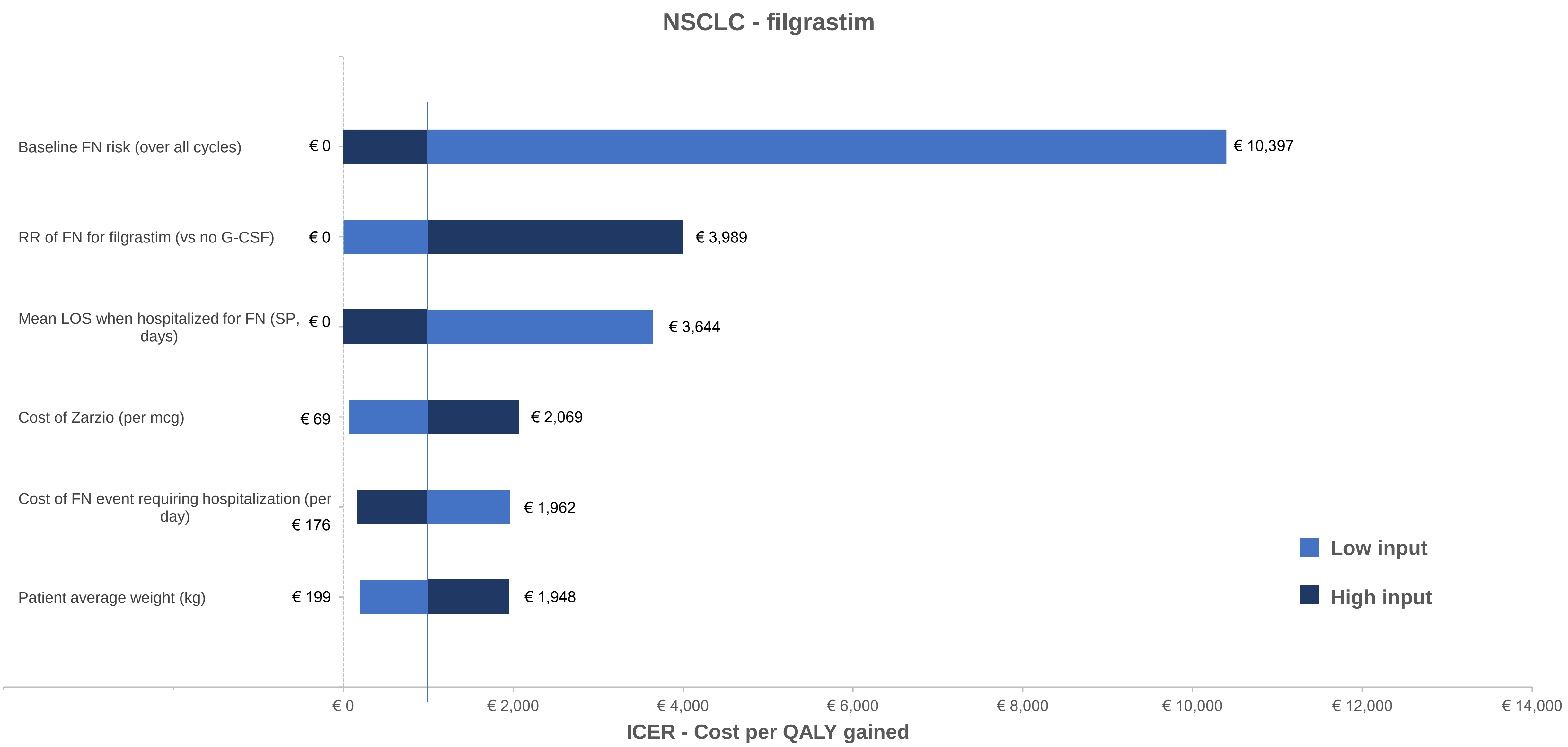
NSCLC - filgrastim
Baseline FN risk (over all cycles)
€ 0
€ 10,397
RR of FN for filgrastim (vs no G-CSF)
€ 0
€ 3,989
Mean LOS when hospitalized for FN (SP, days)
€ 0
€ 3,644
Cost of Zarzio (per mcg)
€ 69
€ 2,069
Cost of FN event requiring hospitalization (per day)
€ 176
€ 1,962
Patient average weight (kg)
€ 199
€ 1,948
Low input
High input
€ 0
€ 2,000
€ 4,000
€ 6,000
€ 8,000
€ 10,000
€ 12,000
€ 14,000
ICER - Cost per QALY gained

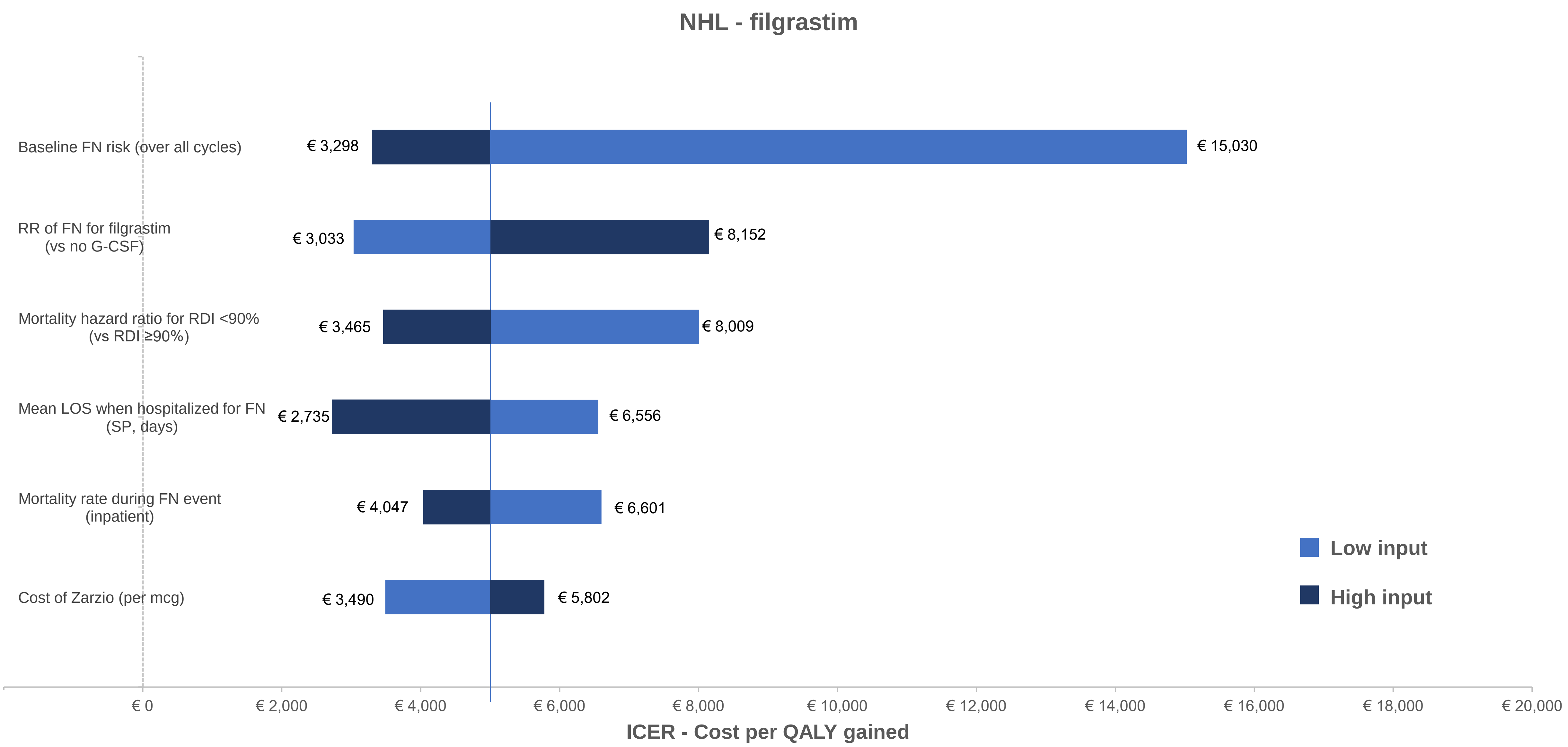

NHL - filgrastim
Baseline FN risk (over all cycles)
RR of FN for filgrastim (vs no G-CSF)
Mortality hazard ratio for RDI <90% (vs RDI ≥90%)
Mean LOS when hospitalized for FN (SP, days)
Mortality rate during FN event (inpatient)
Cost of Zarzio (per mcg)
€ 3,298
€ 15,030
€ 3,033
€ 8,152
€ 3,465
€ 8,009
€ 2,735
€ 6,556
€ 4,047
€ 6,601
€ 3,490
€ 5,802
Low input
High input
€ 0
€ 2,000
€ 4,000
€ 6,000
€ 8,000
€ 10,000
€ 12,000
€ 14,000
€ 16,000
€ 18,000
€ 20,000
ICER - Cost per QALY gained

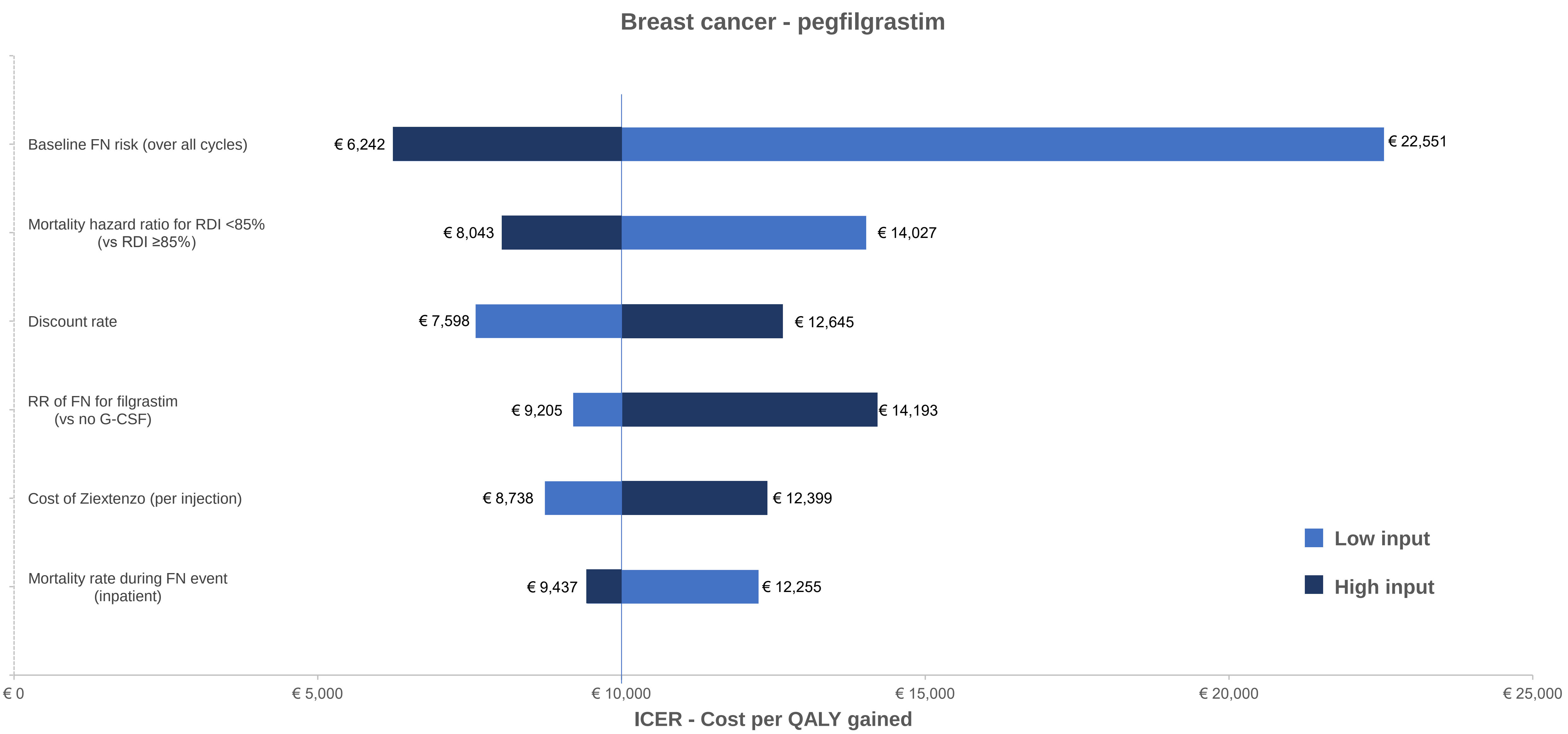

Breast cancer - pegfilgrastim
Baseline FN risk (over all cycles)
Mortality hazard ratio for RDI <85% (vs RDI ≥85%)
Discount rate
RR of FN for filgrastim (vs no G-CSF)
Cost of Ziextenzo (per injection)
Mortality rate during FN event (inpatient)
€ 6,242
€ 22,551
€ 8,043
€ 14,027
€ 7,598
€ 12,645
€ 9,205
€ 14,193
€ 8,738
€ 12,399
€ 9,437
€ 12,255
Low input
High input
€ 0
€ 5,000
€ 10,000
€ 15,000
€ 20,000
€ 25,000
ICER - Cost per QALY gained

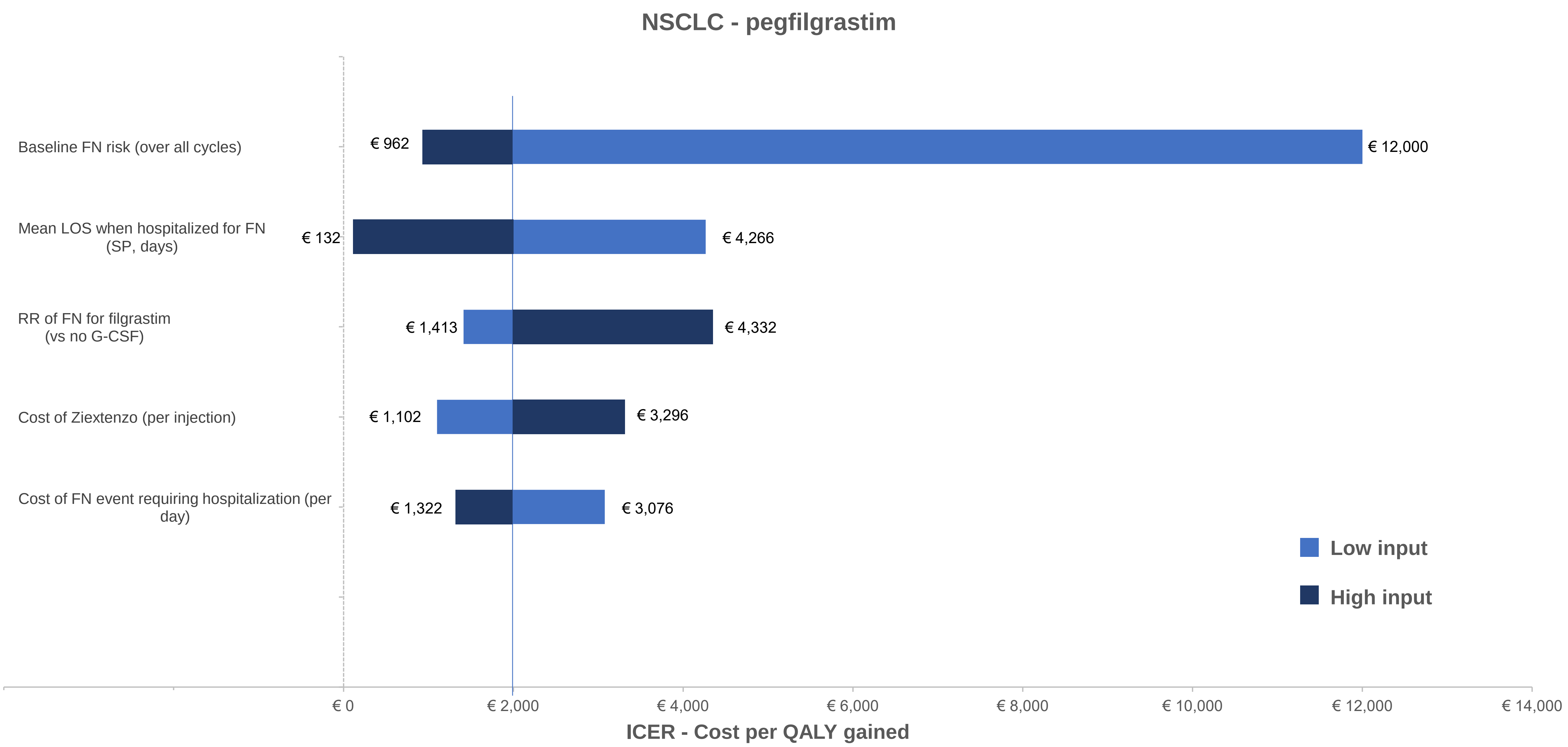
NSCLC - pegfilgrastim
Baseline FN risk (over all cycles)
Mean LOS when hospitalized for FN (SP, days)
RR of FN for filgrastim (vs no G-CSF)
Cost of Ziextenzo (per injection)
Cost of FN event requiring hospitalization (per day)
€ 962
€ 12,000
€ 132
€ 4,266
€ 1,413
€ 4,332
€ 1,102
€ 3,296
€ 1,322
€ 3,076
Low input
High input
€ 0
€ 2,000
€ 4,000
€ 6,000
€ 8,000
€ 10,000
€ 12,000
€ 14,000
ICER - Cost per QALY gained

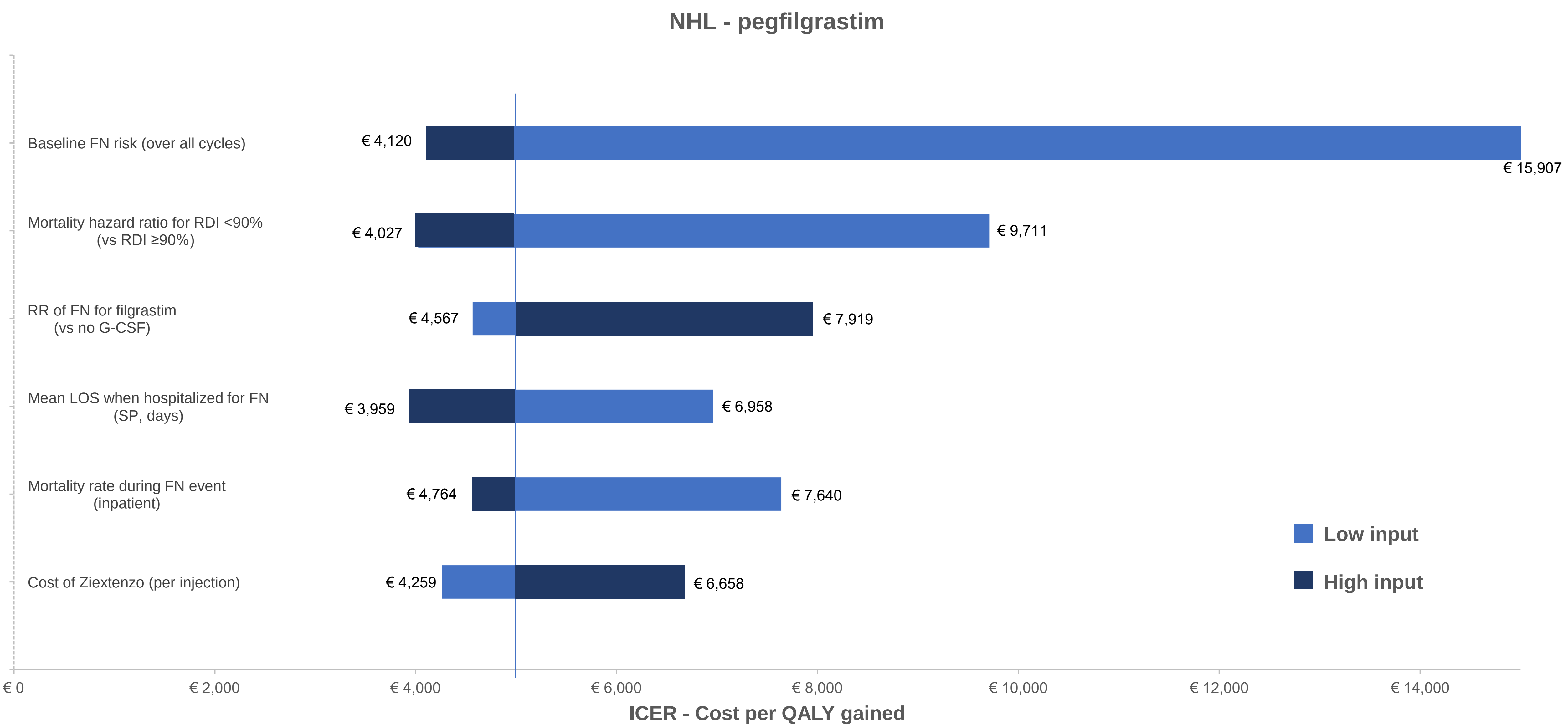

NHL - pegfilgrastim
Baseline FN risk (over all cycles)
Mortality hazard ratio for RDI <90% (vs RDI ≥90%)
RR of FN for filgrastim (vs no G-CSF)
Mean LOS when hospitalized for FN (SP, days)
Mortality rate during FN event (inpatient)
Cost of Ziextenzo (per injection)
€ 4,120
€ 15,907
€ 4,027
€ 9,711
€ 4,567
€ 7,919
€ 3,959
€ 6,958
€ 4,764
€ 7,640
€ 4,259
€ 6,658
Low input
High input
€ 0
€ 2,000
€ 4,000
€ 6,000
€ 8,000
€ 10,000
€ 12,000
€ 14,000
ICER - Cost per QALY gained
