# Supplementary material for: Cost-effectiveness of granulocyte colony-stimulating factors (G-CSFs) for the prevention of febrile neutropenia (FN) in patients with cancer
Source: Support Care Cancer. 2023 Sep 20;31(10):581. doi: 10.1007/s00520-023-08043-4 (PMC10511548; doi:10.1007/s00520-023-08043-4)
Supplement: Supplementary file 3 — (PDF 218 kb) [file 520_2023_8043_MOESM3_ESM.pdf]

Title: Cost -effectiveness of granulocyte colony stimulating factors (G-CSFs) for the prevention of febrile neutropenia (FN) in patients with cancer

Authors: Matti S. Aapro[a], MD, Stephen Chaplin[b], BSc, Paul Cornes[c], BM; Sebastian Howe[d], PhD, Hartmut Link[e], MD, PhD, Natalia Koptelova[d], MD, Andrea Mehl[d], MBA, MPHEco, Mario Di Palma[f], MD, Bridgette Kanz Schroader[g], PharmD, MPA, BCOP, Robert Terkola[h,i], PhD, MSc, aHPh.

Affiliations: [a] Genolier Cancer Center, Genolier, Switzerland; [b] Xcenda UK, York; [c] Comparative Outcomes Group, UK; [d] Sandoz International GmbH, Holzkirchen, Germany; [e] Private Practice Hematology Oncology Kaiserslautern, Germany; [f] Gustave Roussy, Paris-Saclay University, Villejuif, France; [g] Xcenda LLC, Carrollton, TX, US; [h] Institute of Science in Healthy Ageing & HealthcarE (SHARE, University Medical Center Groningen (UMCG, University of Groningen, The Netherlands); [i] Department of Pharmacotherapy and Translational Research, University of Florida -College of Pharmacy, Gainesville, USA

Corresponding author:

Dr Sebastian Howe

Sandoz International GmbH

Industriestr. 18

D-83607 Holzkirchen

Germany

Telephone: +49 8024 4763508

Email address: sebastian.howe@sandoz.com

Online Resource 3: France OWSA

Note: OWSA results for filgrastim in NSCLC are not shown as PP dominated SP.

Abbreviations: FN – febrile neutropenia; G-CSF – granulocyte colony stimulating factor; ICER – incremental cost-effectiveness ratio; kg – kilogram; LOS – length of stay; mcg – microgram; NHL – non-Hodgkin's lymphoma; NSCLC – non-small cell lung cancer; OWSA – one-way sensitivity analysis; PP – primary prophylaxis; QALY – quality-adjusted life year; RDI – relative dose intensity; SP – secondary prophylaxis.

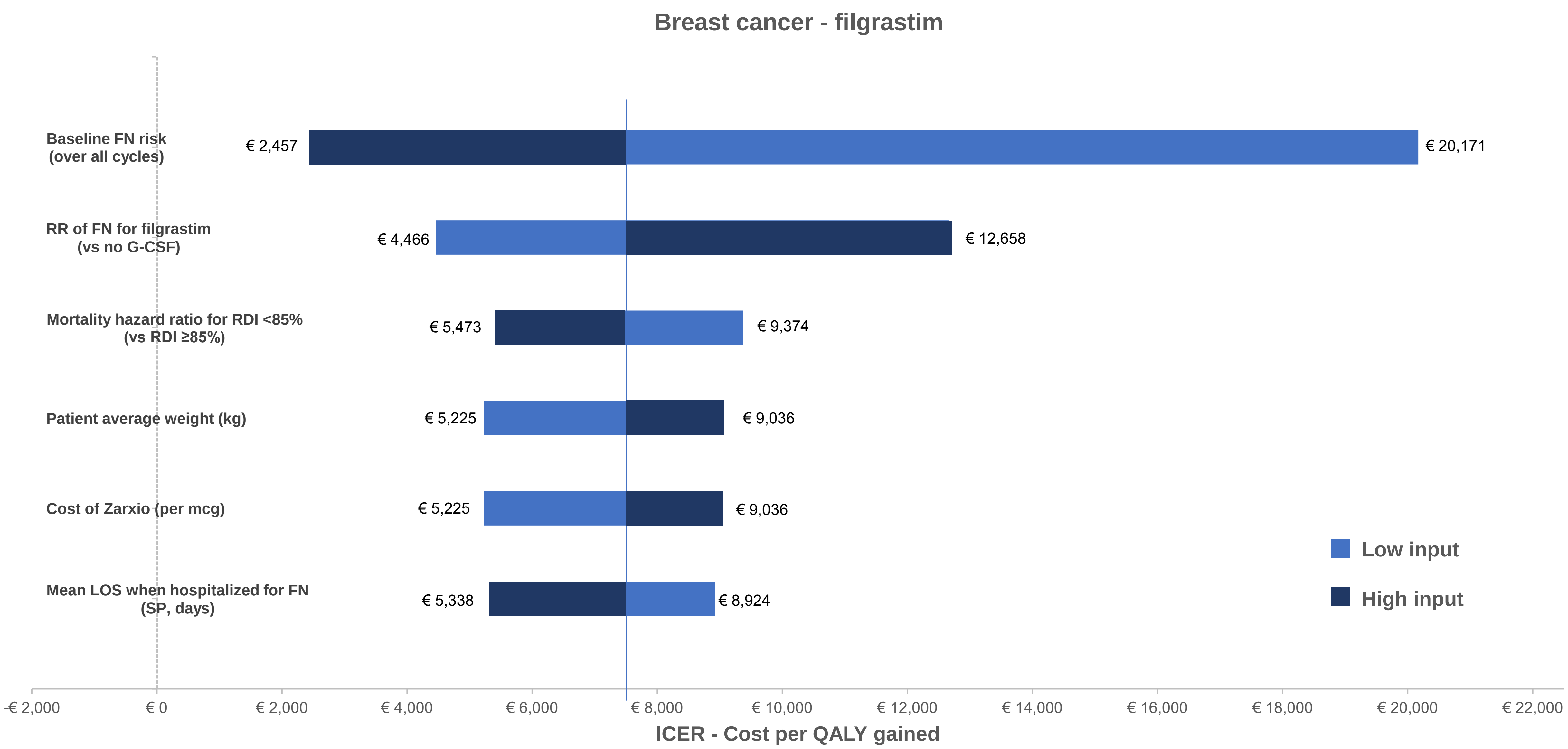
Breast cancer - filgrastim
Baseline FN risk (over all cycles)
€ 2,457
€ 20,171
RR of FN for filgrastim (vs no G-CSF)
€ 4,466
€ 12,658
Mortality hazard ratio for RDI <85% (vs RDI ≥85%)
€ 5,473
€ 9,374
Patient average weight (kg)
€ 5,225
€ 9,036
Cost of Zarxio (per mcg)
€ 5,225
€ 9,036
Mean LOS when hospitalized for FN (SP, days)
€ 5,338
€ 8,924
Low input
High input
-€ 2,000
€ 0
€ 2,000
€ 4,000
€ 6,000
€ 8,000
€ 10,000
€ 12,000
€ 14,000
€ 16,000
€ 18,000
€ 20,000
€ 22,000
ICER - Cost per QALY gained

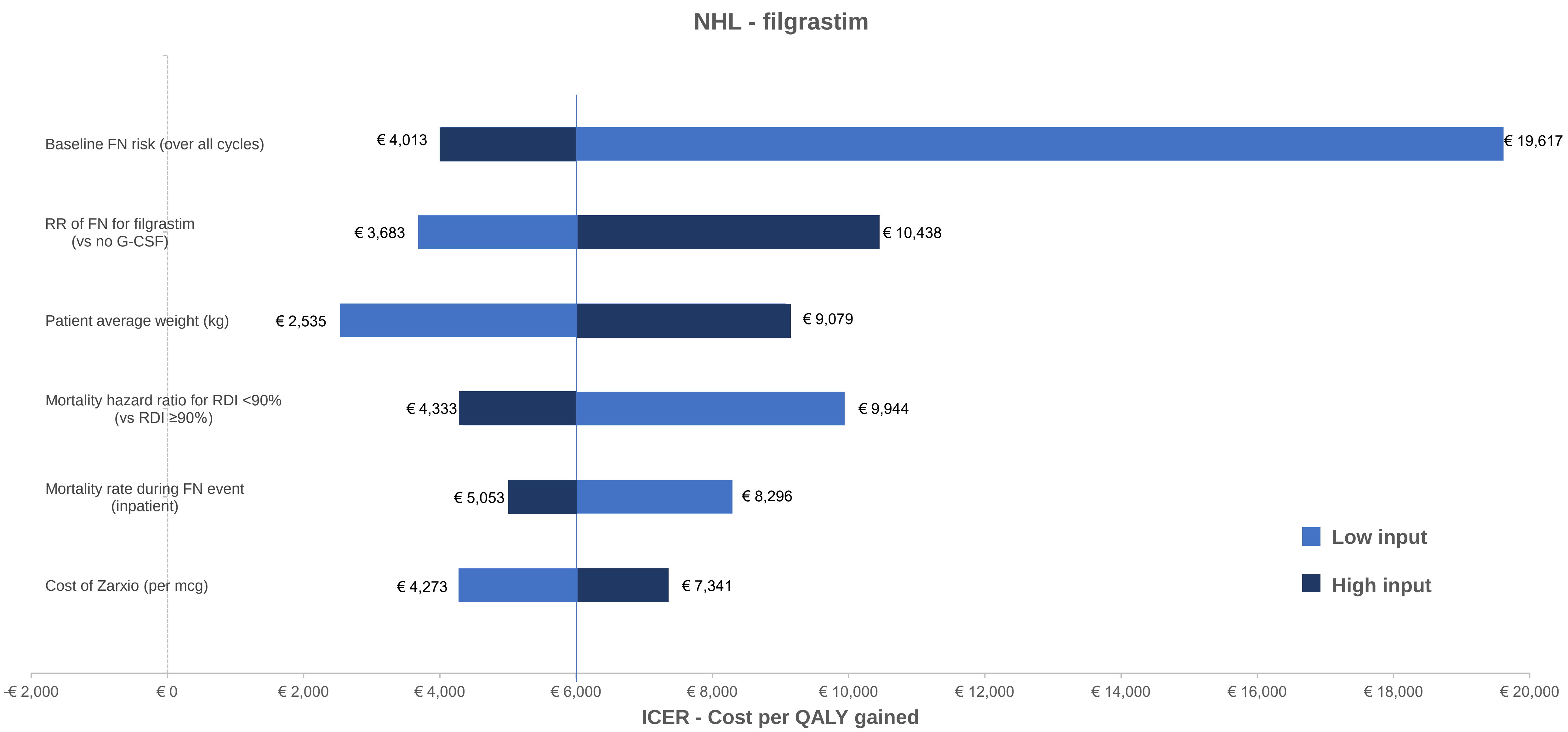

NHL - filgrastim
Baseline FN risk (over all cycles)
RR of FN for filgrastim (vs no G-CSF)
Patient average weight (kg)
Mortality hazard ratio for RDI <90% (vs RDI ≥90%)
Mortality rate during FN event (inpatient)
Cost of Zarxio (per mcg)
€ 4,013
€ 19,617
€ 3,683
€ 10,438
€ 2,535
€ 9,079
€ 4,333
€ 9,944
€ 5,053
€ 8,296
€ 4,273
€ 7,341
Low input
High input
-€ 2,000
€ 0
€ 2,000
€ 4,000
€ 6,000
€ 8,000
€ 10,000
€ 12,000
€ 14,000
€ 16,000
€ 18,000
€ 20,000
ICER - Cost per QALY gained

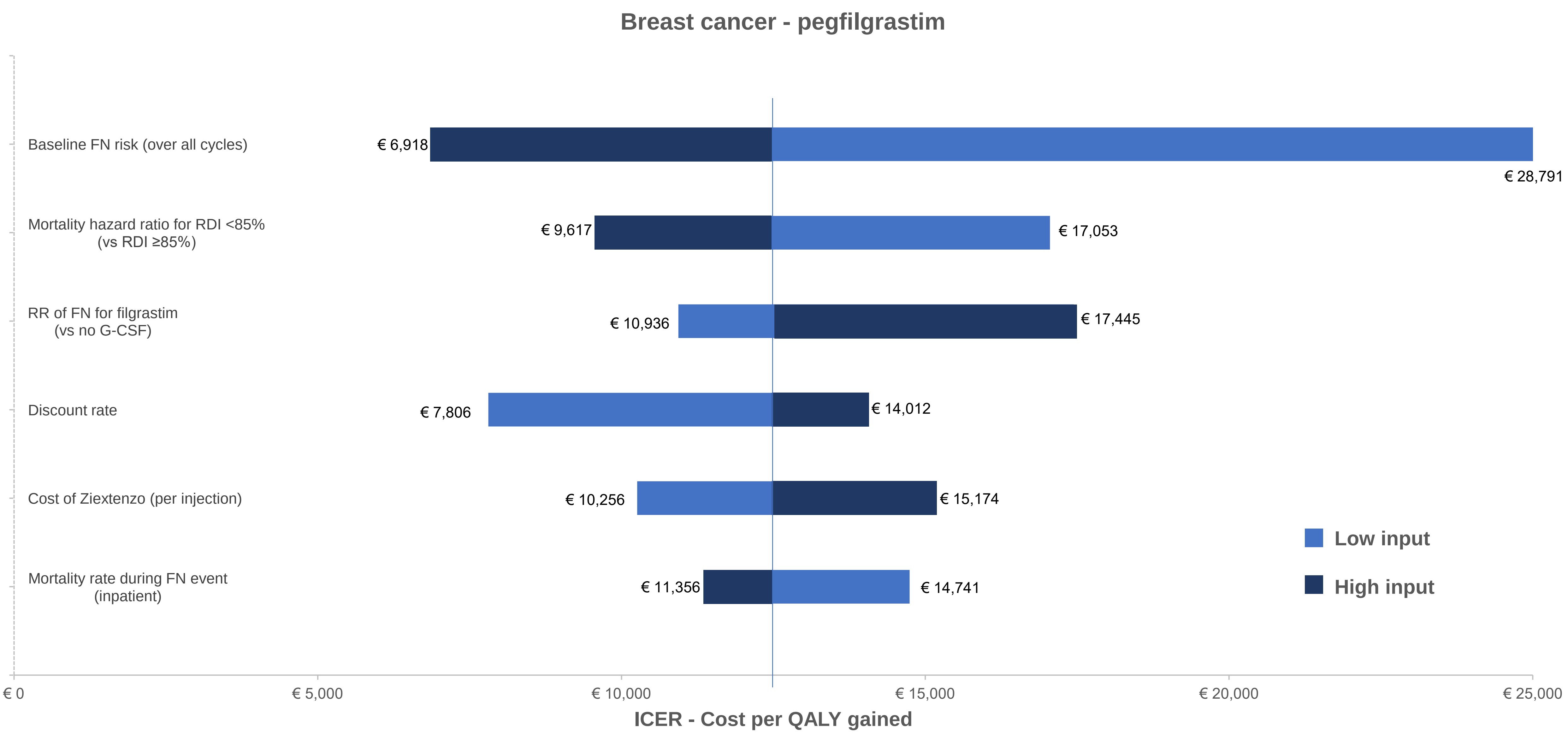
Breast cancer - pegfilgrastim
Baseline FN risk (over all cycles)
€ 6,918
€ 28,791
Mortality hazard ratio for RDI <85% (vs RDI ≥85%)
€ 9,617
€ 17,053
RR of FN for filgrastim (vs no G-CSF)
€ 10,936
€ 17,445
Discount rate
€ 7,806
€ 14,012
Cost of Ziextenzo (per injection)
€ 10,256
€ 15,174
Mortality rate during FN event (inpatient)
€ 11,356
€ 14,741
Low input
High input
€ 0
€ 5,000
€ 10,000
€ 15,000
€ 20,000
€ 25,000
ICER - Cost per QALY gained

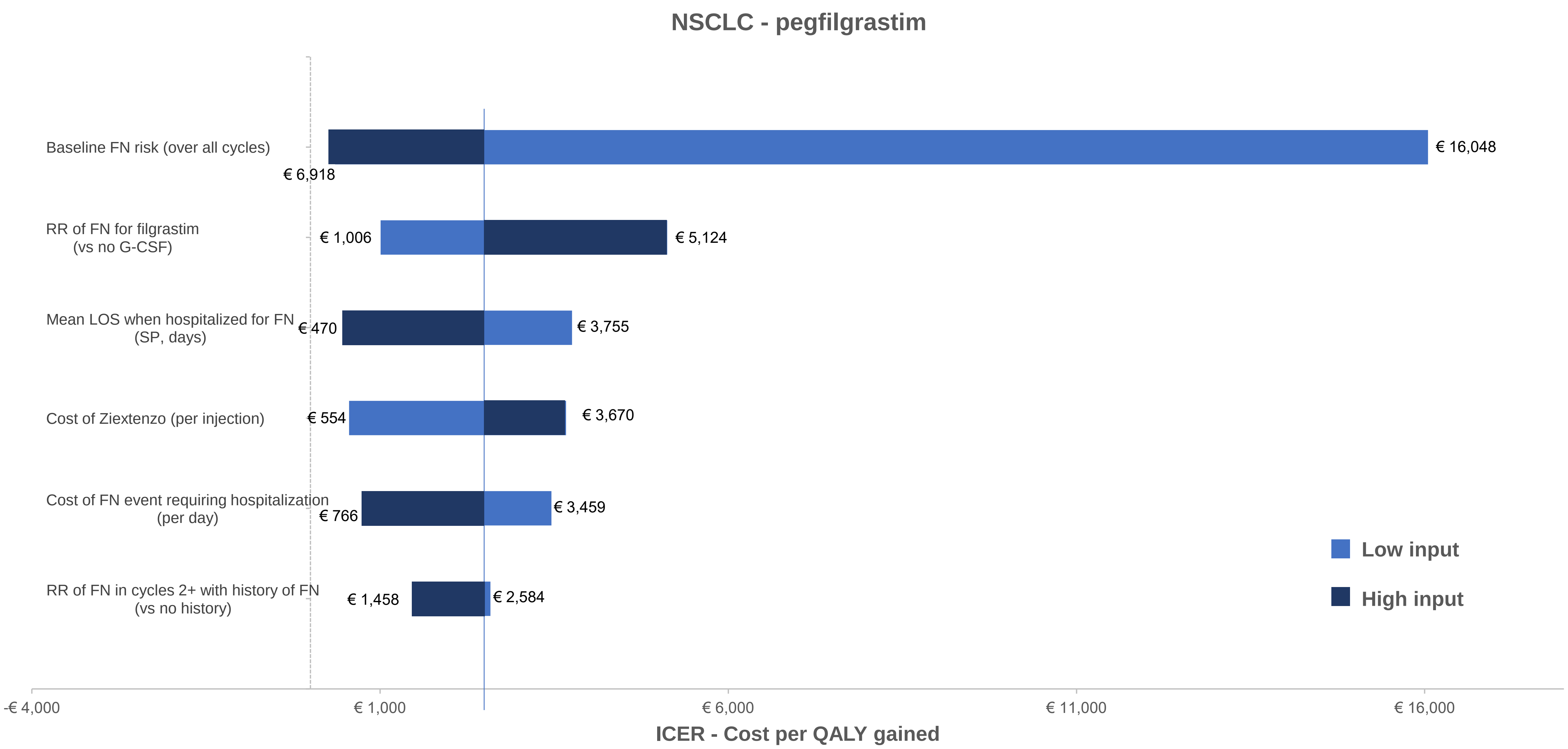

NSCLC - pegfilgrastim
Baseline FN risk (over all cycles)
€ 6,918
€ 16,048
RR of FN for filgrastim
(vs no G-CSF)
€ 1,006
€ 5,124
Mean LOS when hospitalized for FN
(SP, days)
€ 470
€ 3,755
Cost of Ziextenzo (per injection)
€ 554
€ 3,670
Cost of FN event requiring hospitalization
(per day)
€ 766
€ 3,459
RR of FN in cycles 2+ with history of FN
(vs no history)
€ 1,458
€ 2,584
Low input
High input
-€ 4,000
€ 1,000
€ 6,000
€ 11,000
€ 16,000
ICER - Cost per QALY gained

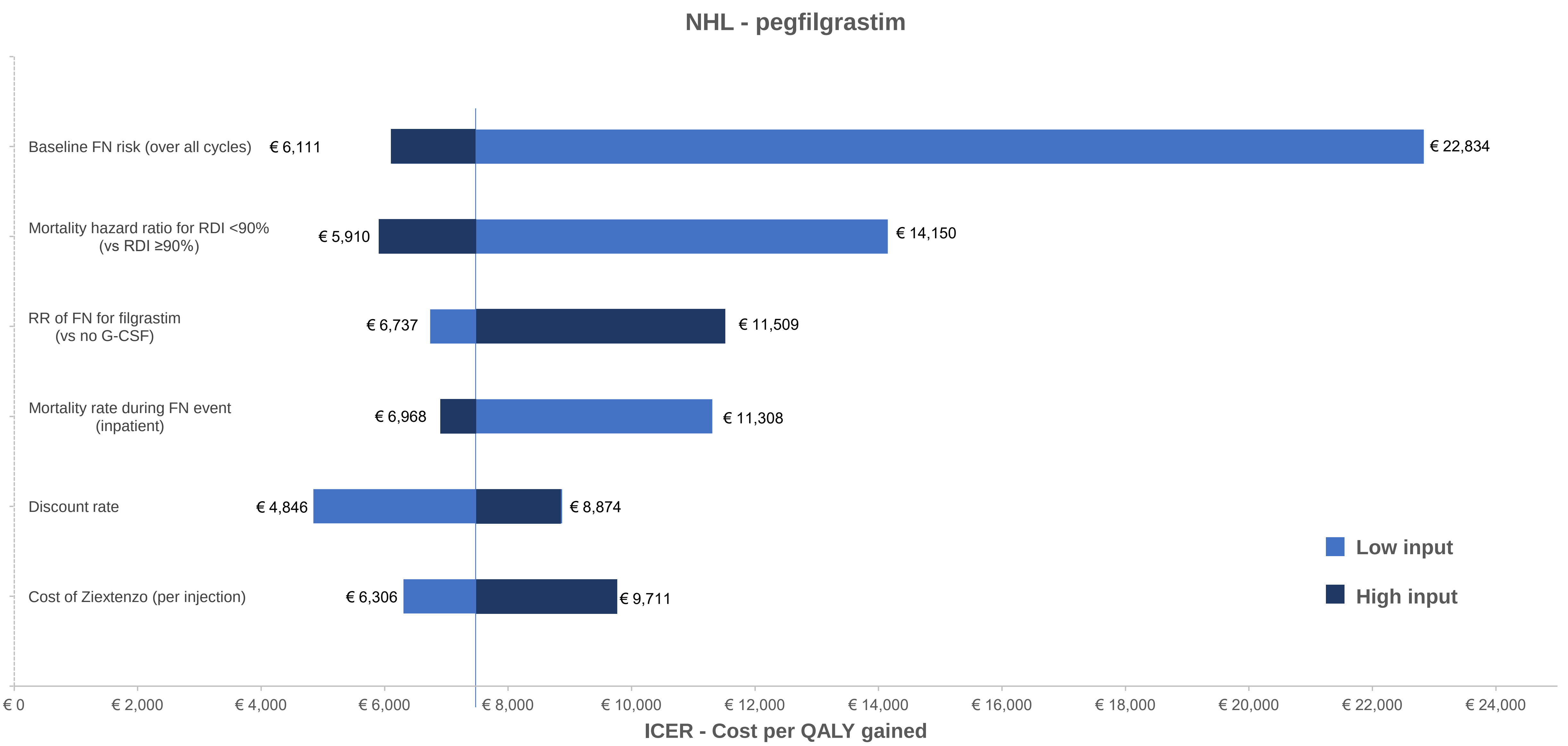
NHL - pegfilgrastim
Baseline FN risk (over all cycles)
€ 6,111
€ 22,834
Mortality hazard ratio for RDI <90% (vs RDI ≥90%)
€ 5,910
€ 14,150
RR of FN for filgrastim (vs no G-CSF)
€ 6,737
€ 11,509
Mortality rate during FN event (inpatient)
€ 6,968
€ 11,308
Discount rate
€ 4,846
€ 8,874
Cost of Ziextenzo (per injection)
€ 6,306
€ 9,711
Low input
High input
€ 0
€ 2,000
€ 4,000
€ 6,000
€ 8,000
€ 10,000
€ 12,000
€ 14,000
€ 16,000
€ 18,000
€ 20,000
€ 22,000
€ 24,000
ICER - Cost per QALY gained
