# Supplementary material for: Cost-effectiveness of granulocyte colony-stimulating factors (G-CSFs) for the prevention of febrile neutropenia (FN) in patients with cancer
Source: Support Care Cancer. 2023 Sep 20;31(10):581. doi: 10.1007/s00520-023-08043-4 (PMC10511548; doi:10.1007/s00520-023-08043-4)
Supplement: Supplementary file 4 — (PDF 221 kb) [file 520_2023_8043_MOESM4_ESM.pdf]

Title: Cost -effectiveness of granulocyte colony stimulating factors (G-CSFs) for the prevention of febrile neutropenia (FN) in patients with cancer

Authors: Matti S. Aapro[a], MD, Stephen Chaplin[b], BSc, Paul Cornes[c], BM; Sebastian Howe[d], PhD, Hartmut Link[e], MD, PhD, Natalia Koptelova[d], MD, Andrea Mehl[d], MBA, MPHEco, Mario Di Palma[f], MD, Bridgette Kanz Schroader[g], PharmD, MPA, BCOP, Robert Terkola[h,i], PhD, MSc, aHPh.

Affiliations: [a] Genolier Cancer Center, Genolier, Switzerland; [b] Xcenda UK, York; [c] Comparative Outcomes Group, UK; [d] Sandoz International GmbH, Holzkirchen, Germany; [e] Private Practice Hematology Oncology Kaiserslautern, Germany; [f] Gustave Roussy, Paris-Saclay University, Villejuif, France; [g] Xcenda LLC, Carrollton, TX, US; [h] Institute of Science in Healthy Ageing & HealthcarE (SHARE, University Medical Center Groningen (UMCG, University of Groningen, The Netherlands); [i] Department of Pharmacotherapy and Translational Research, University of Florida -College of Pharmacy, Gainesville, USA

Corresponding author:

Dr Sebastian Howe

Sandoz International GmbH

Industriestr. 18

D-83607 Holzkirchen

Germany

Telephone: +49 8024 4763508

Email address: sebastian.howe@sandoz.com

Online Resource 4: Germany OWSA

Abbreviations: FN – febrile neutropenia; G-CSF – granulocyte colony stimulating factor; ICER – incremental cost-effectiveness ratio; kg – kilogram; LOS – length of stay; mcg – microgram; NHL – non-Hodgkin's lymphoma; NSCLC – non-small cell lung cancer; OWSA – one-way sensitivity analysis; QALY – quality-adjusted life year; RDI – relative dose intensity; SP – secondary prophylaxis.

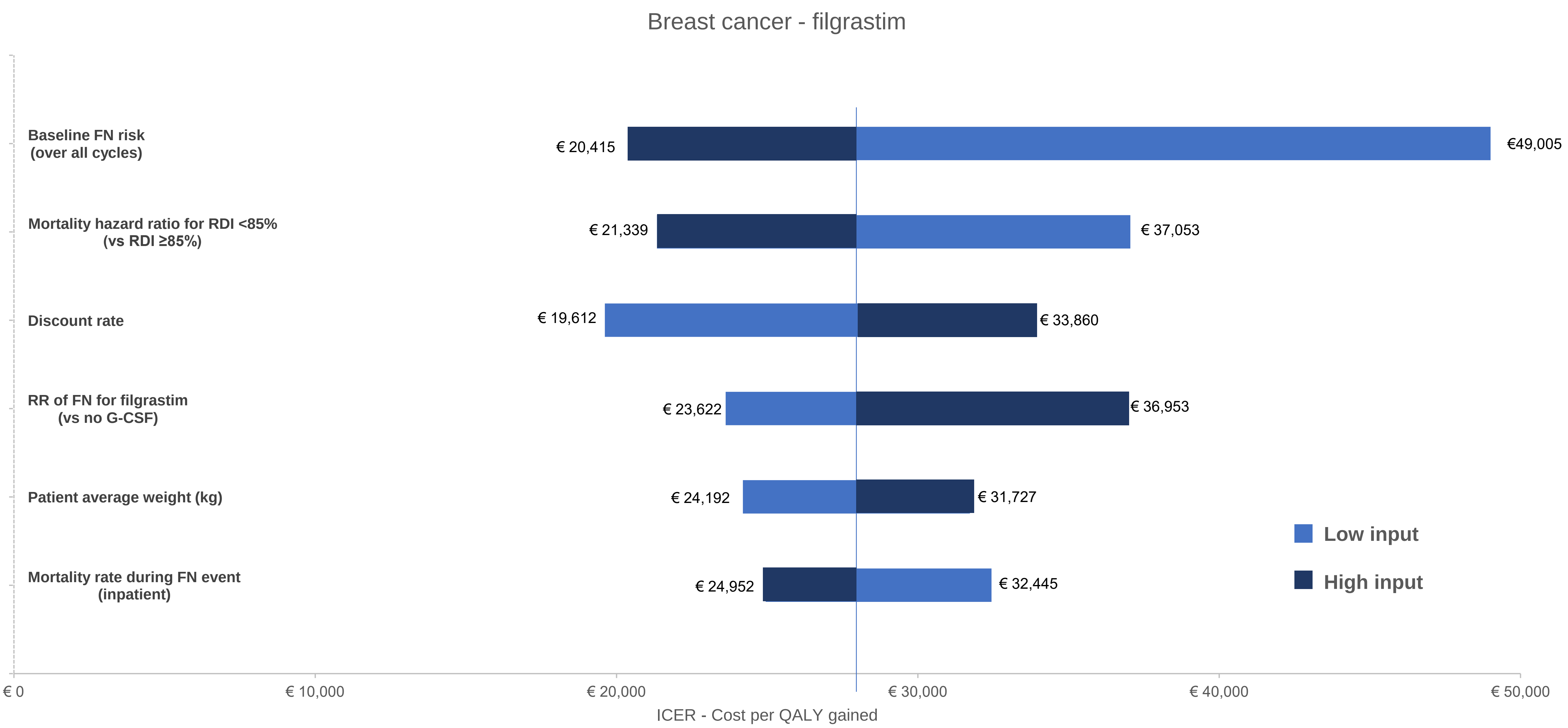
Breast cancer - filgrastim
Baseline FN risk
(over all cycles)
€ 20,415
€49,005
Mortality hazard ratio for RDI <85%
(vs RDI ≥85%)
€ 21,339
€ 37,053
Discount rate
€ 19,612
€ 33,860
RR of FN for filgrastim
(vs no G-CSF)
€ 23,622
€ 36,953
Patient average weight (kg)
€ 24,192
€ 31,727
Mortality rate during FN event
(inpatient)
€ 24,952
€ 32,445
Low input
High input
€ 0
€ 10,000
€ 20,000
€ 30,000
€ 40,000
€ 50,000
ICER - Cost per QALY gained

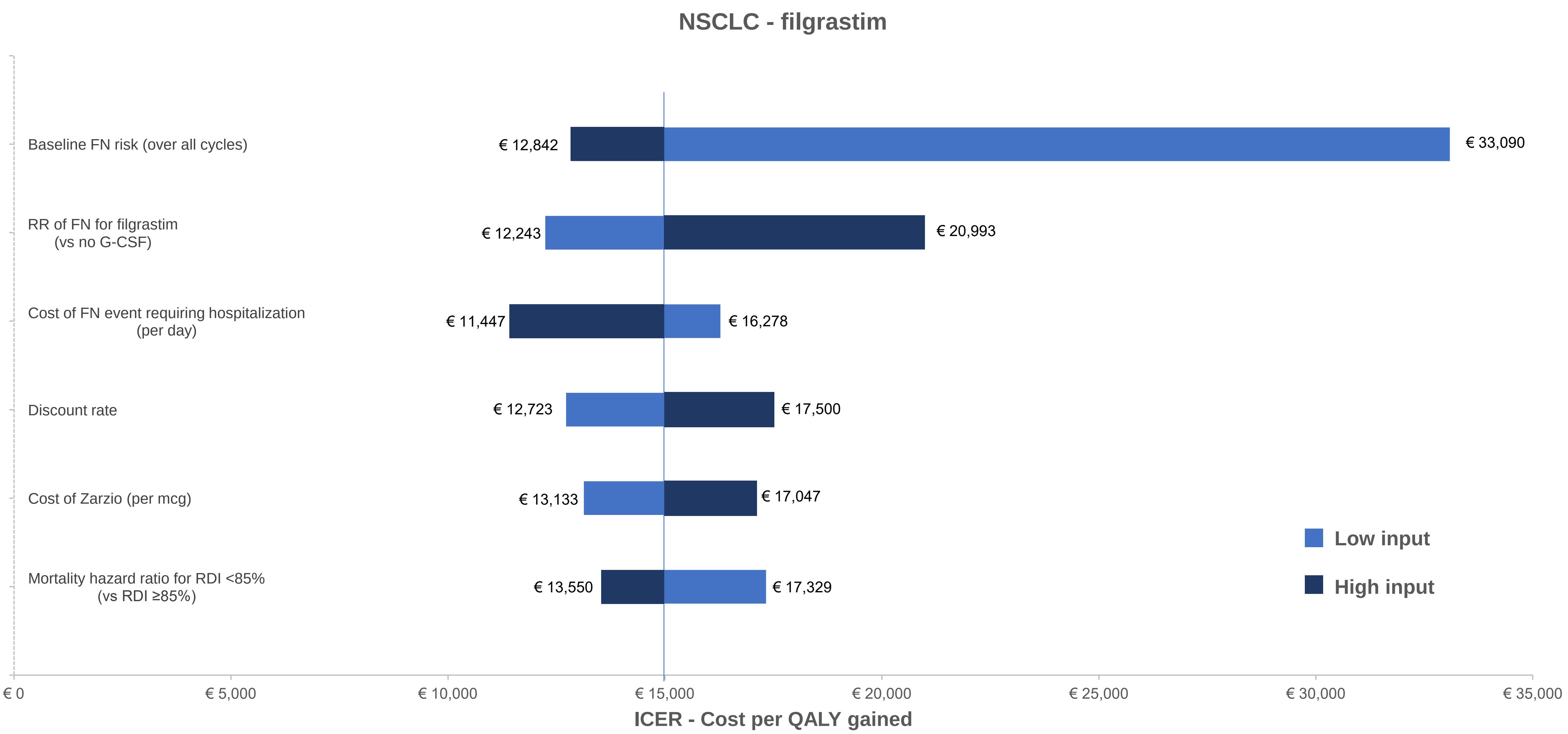
NSCLC - filgrastim
Baseline FN risk (over all cycles)
€ 12,842
€ 33,090
RR of FN for filgrastim
(vs no G-CSF)
€ 12,243
€ 20,993
Cost of FN event requiring hospitalization
(per day)
€ 11,447
€ 16,278
Discount rate
€ 12,723
€ 17,500
Cost of Zarzio (per mcg)
€ 13,133
€ 17,047
Mortality hazard ratio for RDI <85%
(vs RDI ≥85%)
€ 13,550
€ 17,329
Low input
High input
€ 0
€ 5,000
€ 10,000
€ 15,000
€ 20,000
€ 25,000
€ 30,000
€ 35,000
ICER - Cost per QALY gained

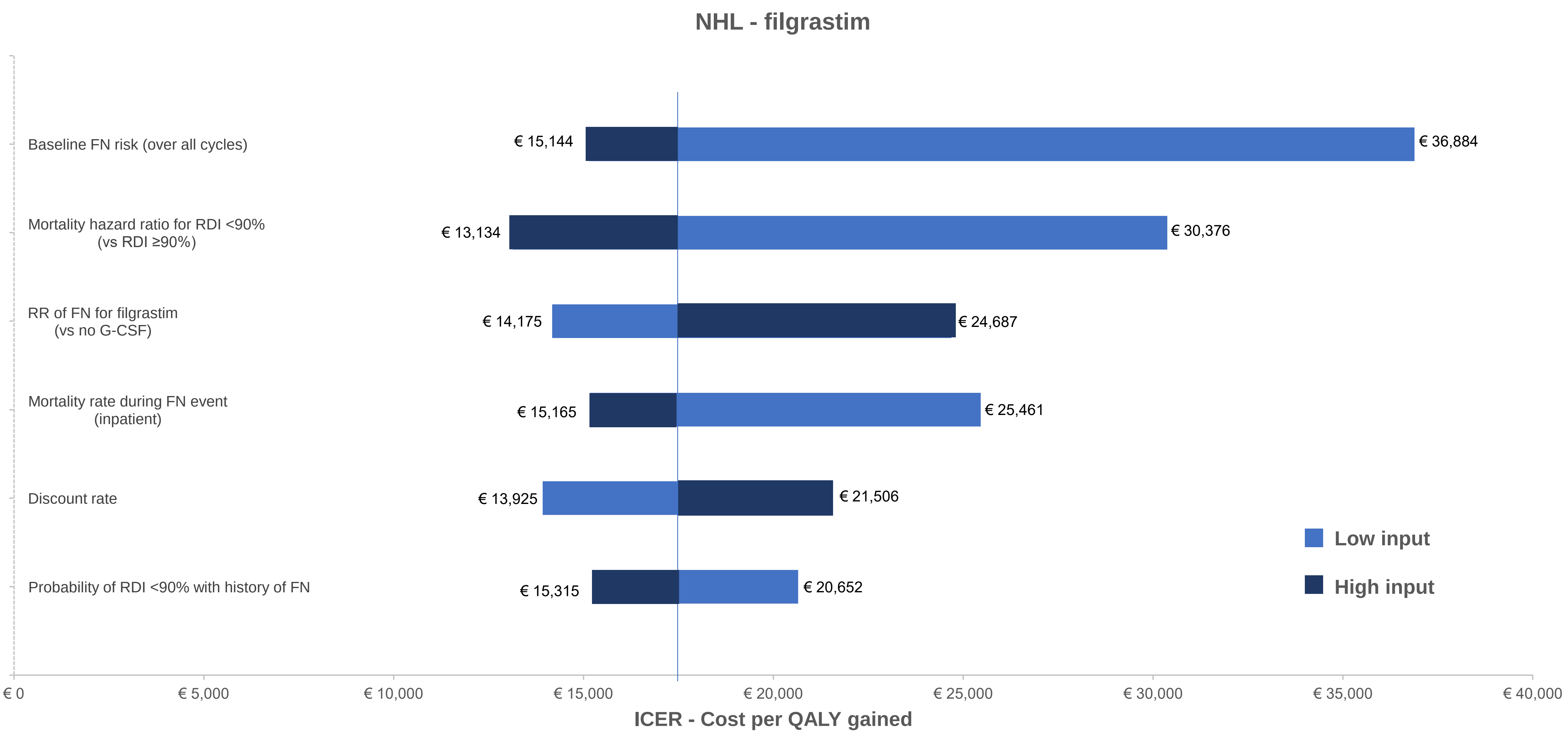
NHL - filgrastim
Baseline FN risk (over all cycles)
Mortality hazard ratio for RDI <90% (vs RDI ≥90%)
RR of FN for filgrastim (vs no G-CSF)
Mortality rate during FN event (inpatient)
Discount rate
Probability of RDI <90% with history of FN
€ 15,144
€ 36,884
€ 13,134
€ 30,376
€ 14,175
€ 24,687
€ 15,165
€ 25,461
€ 13,925
€ 21,506
€ 15,315
€ 20,652
Low input
High input
€ 0
€ 5,000
€ 10,000
€ 15,000
€ 20,000
€ 25,000
€ 30,000
€ 35,000
€ 40,000
ICER - Cost per QALY gained

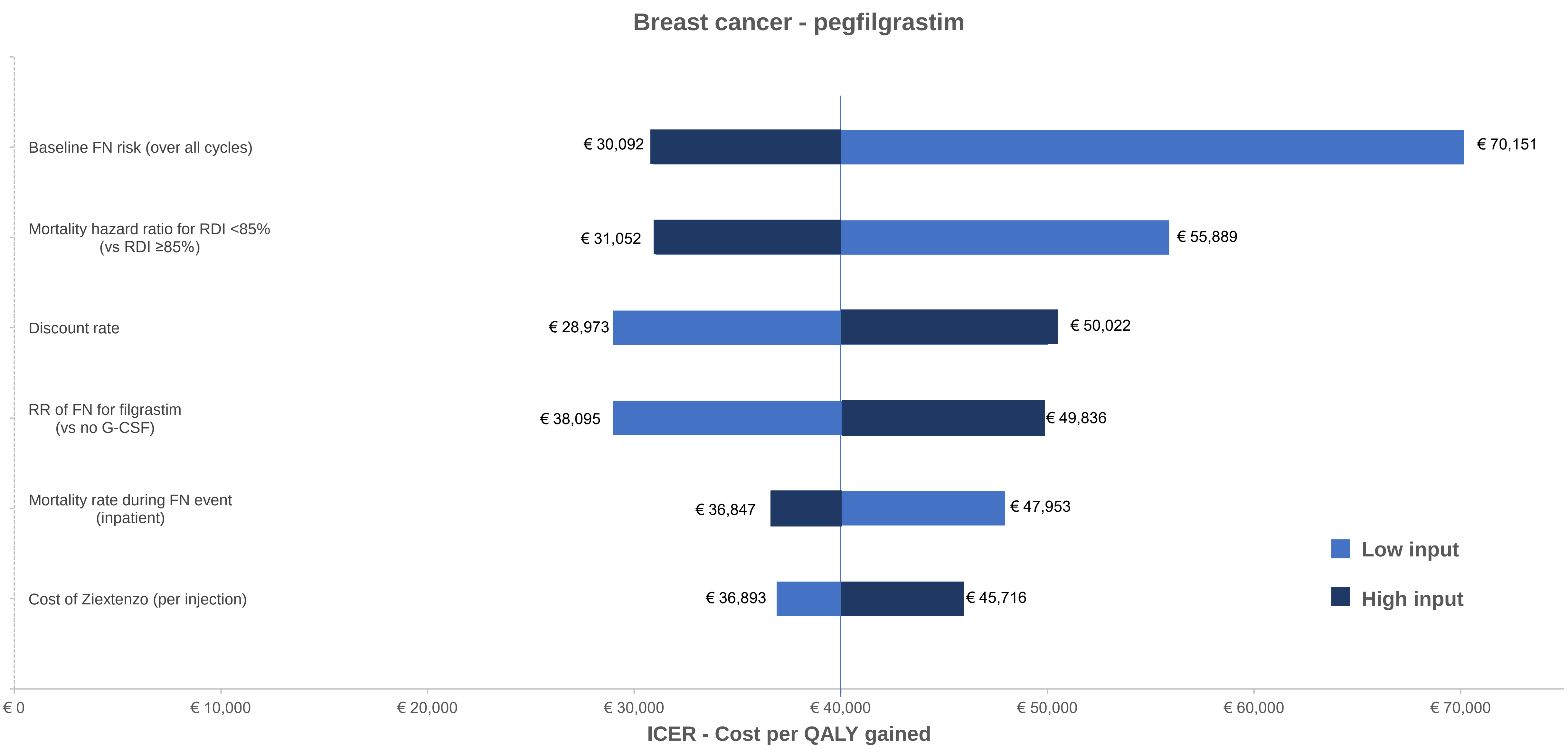
Breast cancer - pegfilgrastim
Baseline FN risk (over all cycles)
€ 30,092
€ 70,151
Mortality hazard ratio for RDI <85% (vs RDI ≥85%)
€ 31,052
€ 55,889
Discount rate
€ 28,973
€ 50,022
RR of FN for filgrastim (vs no G-CSF)
€ 38,095
€ 49,836
Mortality rate during FN event (inpatient)
€ 36,847
€ 47,953
Cost of Ziextenzo (per injection)
€ 36,893
€ 45,716
Low input
High input
€ 0
€ 10,000
€ 20,000
€ 30,000
€ 40,000
€ 50,000
€ 60,000
€ 70,000
ICER - Cost per QALY gained

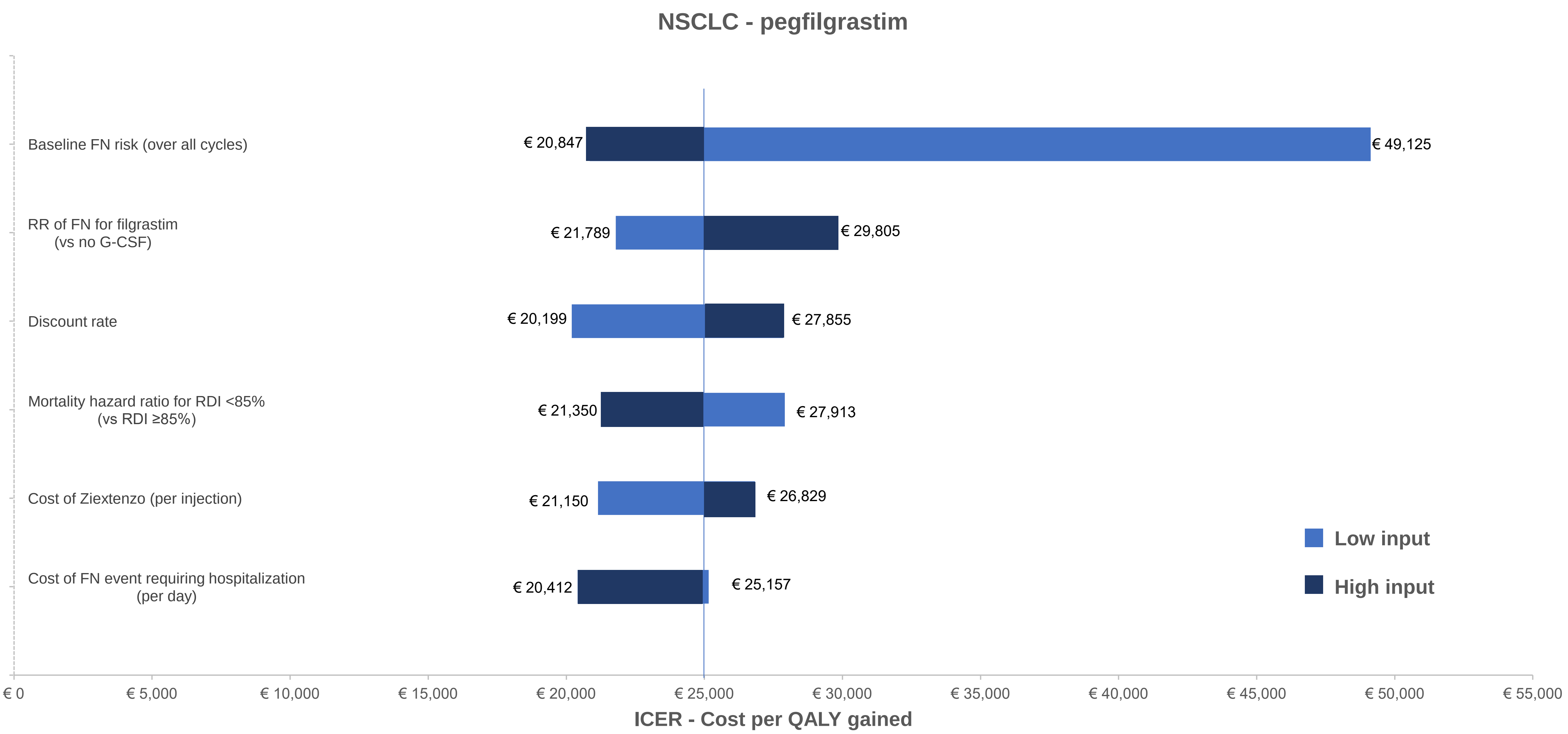
NSCLC - pegfilgrastim
Baseline FN risk (over all cycles)
€ 20,847
€ 49,125
RR of FN for filgrastim
(vs no G-CSF)
€ 21,789
€ 29,805
Discount rate
€ 20,199
€ 27,855
Mortality hazard ratio for RDI <85%
(vs RDI ≥85%)
€ 21,350
€ 27,913
Cost of Ziextenzo (per injection)
€ 21,150
€ 26,829
Cost of FN event requiring hospitalization
(per day)
€ 20,412
€ 25,157
Low input
High input
€ 0
€ 5,000
€ 10,000
€ 15,000
€ 20,000
€ 25,000
€ 30,000
€ 35,000
€ 40,000
€ 45,000
€ 50,000
€ 55,000
ICER - Cost per QALY gained

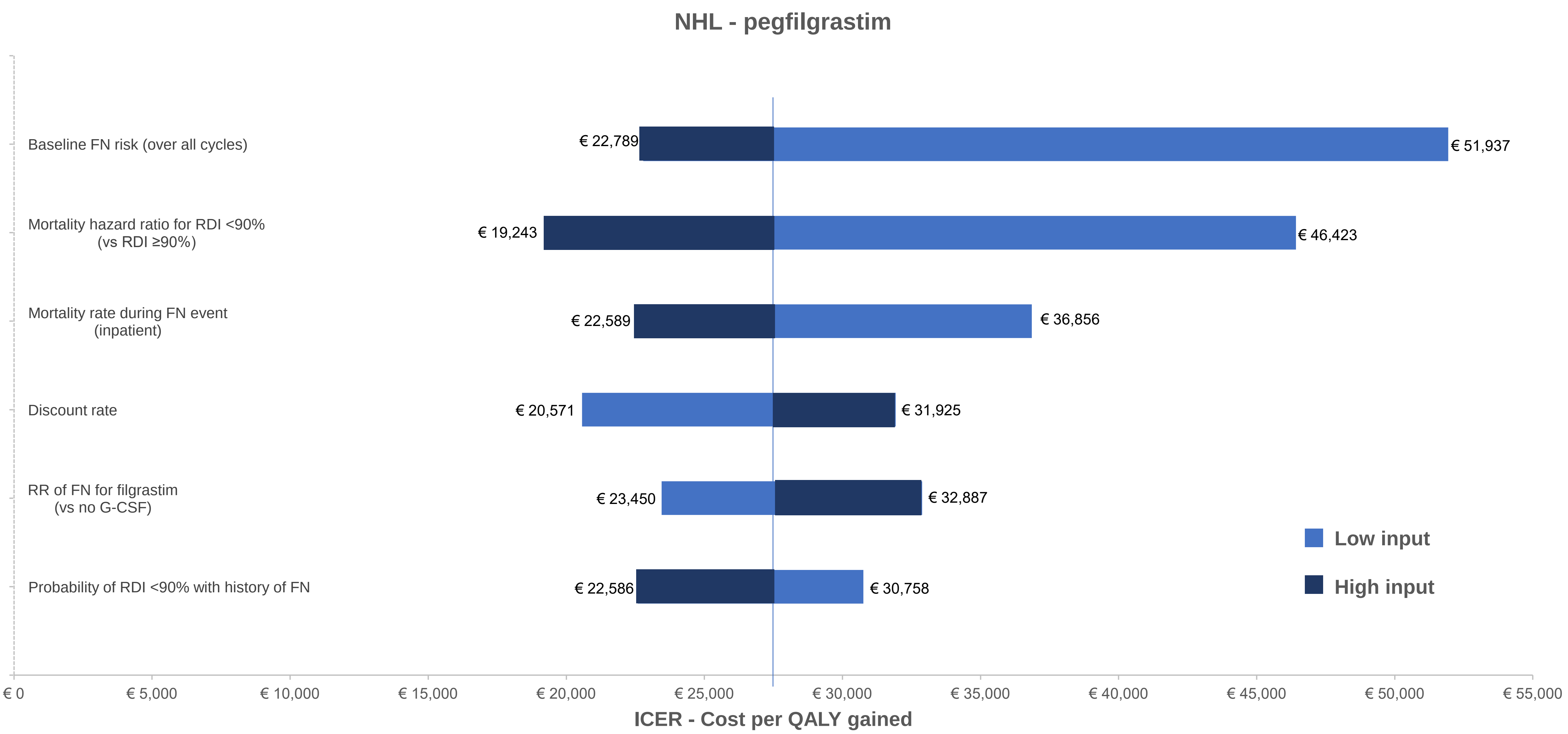
NHL - pegfilgrastim
Baseline FN risk (over all cycles)
Mortality hazard ratio for RDI <90% (vs RDI ≥90%)
Mortality rate during FN event (inpatient)
Discount rate
RR of FN for filgrastim (vs no G-CSF)
Probability of RDI <90% with history of FN
€ 22,789
€ 51,937
€ 19,243
€ 46,423
€ 22,589
€ 36,856
€ 20,571
€ 31,925
€ 23,450
€ 32,887
€ 22,586
€ 30,758
Low input
High input
€ 0
€ 5,000
€ 10,000
€ 15,000
€ 20,000
€ 25,000
€ 30,000
€ 35,000
€ 40,000
€ 45,000
€ 50,000
€ 55,000
ICER - Cost per QALY gained
